# Design, Synthesis, and Evaluation of Lung-Retentive Prodrugs for Extending the Lung Tissue Retention of Inhaled Drugs

**DOI:** 10.1021/acs.jmedchem.2c00416

**Published:** 2022-07-07

**Authors:** Jack Ayre, Joanna M. Redmond, Giovanni Vitulli, Laura Tomlinson, Richard Weaver, Eleonora Comeo, Cynthia Bosquillon, Michael J. Stocks

**Affiliations:** † School of Pharmacy, Biodiscovery Institute, University Park Nottingham, Nottingham NG7 2RD, U.K.; ‡ GSK Medicines Research Centre, Gunnels Wood Road, Stevenage SG1 2NY, U.K.; § XenoGesis Ltd, Discovery Building, BioCity, Pennyfoot Street, Nottingham NG1 1GR, U.K.; ∥ School of Pharmacy, Boots Science Building, University Park Nottingham, Nottingham NG7 2RD, U.K.

## Abstract

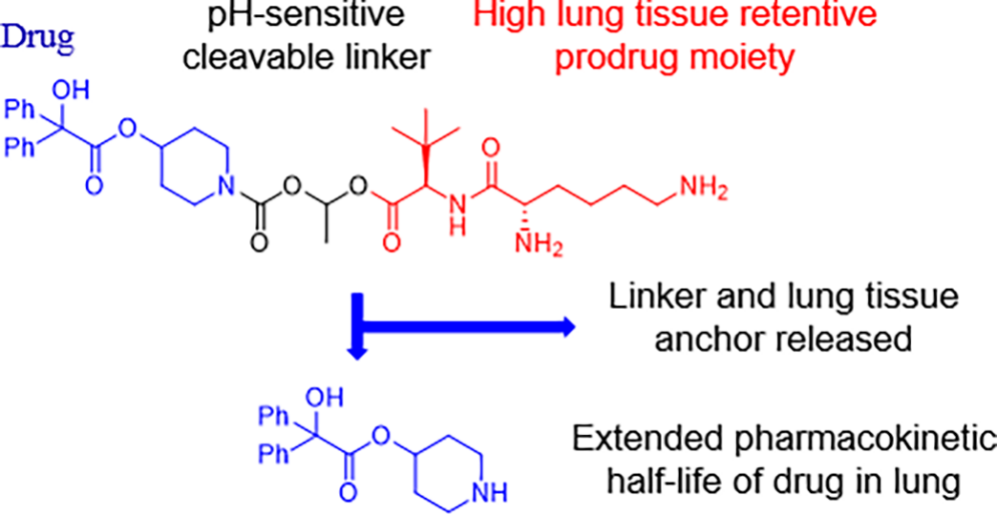

A major limitation of pulmonary delivery is that drugs can exhibit suboptimal pharmacokinetic profiles resulting from rapid elimination from the pulmonary tissue. This can lead to systemic side effects and a short duration of action. A series of dibasic dipeptides attached to the poorly lung-retentive muscarinic M3 receptor antagonist piperidin-4-yl 2-hydroxy-2,2-diphenylacetate (**1**) through a pH-sensitive-linking group have been evaluated. Extensive optimization resulted in 1-(((*R*)-2-((*S*)-2,6-diaminohexanamido)-3,3-dimethylbutanoyl)oxy)ethyl 4-(2-hydroxy-2,2-diphenylacetoxy)piperidine-1-carboxylate (**23**), which combined very good *in vitro* stability and very high rat lung binding. Compound **23** progressed to pharmacokinetic studies in rats, where, at 24 h post dosing in the rat lung, the total lung concentration of **23** was 31.2 μM. In addition, high levels of liberated drug **1** were still detected locally, demonstrating the benefit of this novel prodrug approach for increasing the apparent pharmacokinetic half-life of drugs in the lungs following pulmonary dosing.

## Introduction

Drug inhalation has been successfully exploited as part of the management of respiratory diseases such as asthma and chronic obstructive pulmonary disease (COPD).^1,2^ Recently, emerging literature evidence suggests that the pulmonary delivery route would also be beneficial for the treatment of cancer,^3^ idiopathic pulmonary fibrosis (IPF),^4^ respiratory infections,^5^ and most recently, coronavirus disease (COVID-19).^6^ However, a major limitation of local pulmonary delivery is that, typically, inhaled drugs exhibit a suboptimal pharmacokinetic profile characterized by a high maximum blood concentration (high *C*_max_) that is achieved very shortly post administration (short *T*_max_), resulting from the rapid elimination of compound from the pulmonary tissue. This can lead to systemic side effects and a short duration of action in the lungs.^7^ Therefore, strategies to enhance lung residence time have been explored with the aim of improving the therapeutic index of inhaled therapies as well as decreasing their frequency of administration.^8,9^

**Figure 1:**
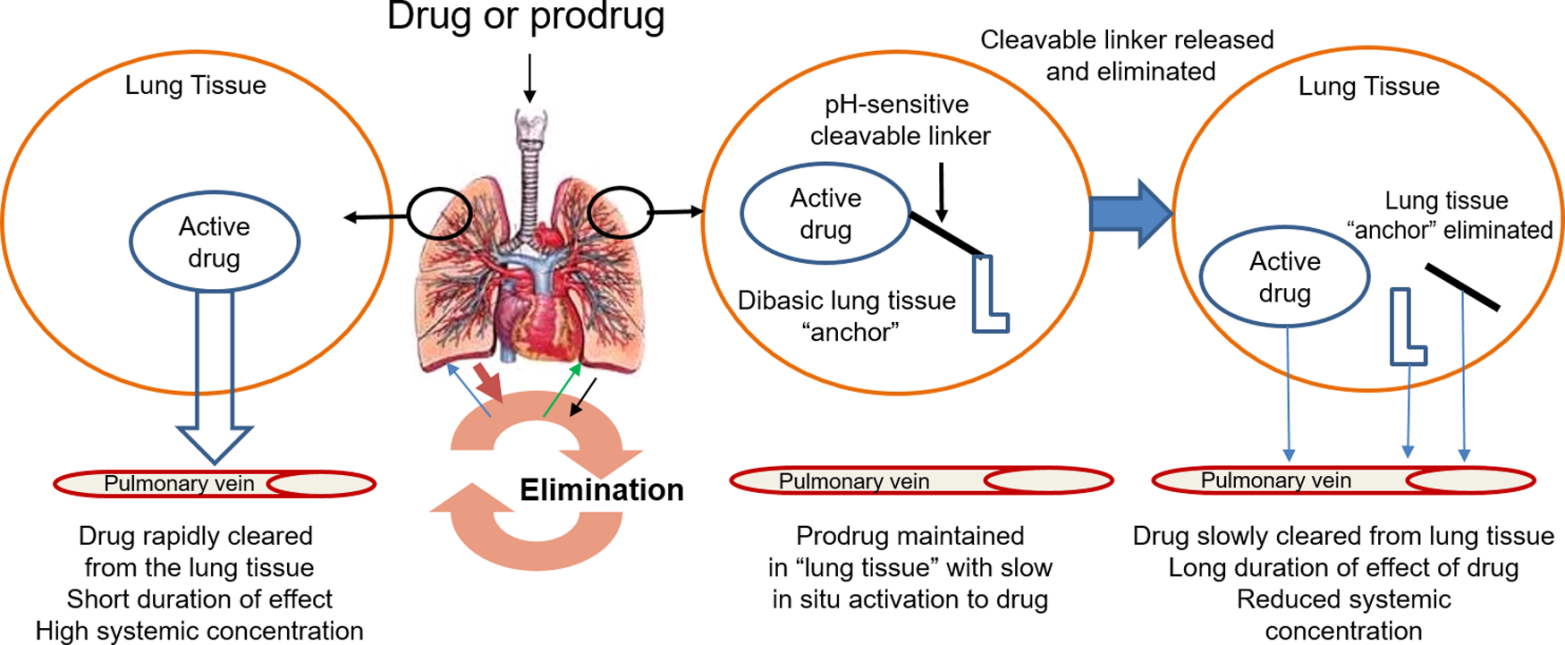
Schematic representation of the novel lung-retentive prodrug concept. This would increase the drug half-life in the lung tissue and reduce its blood concentration; thus, the reduction would be released as acetaldehyde, while the lung tissue anchor would be eliminated by endogenous clearance mechanisms in the lungs, including absorption into the blood, mucociliary clearance, or uptake by macrophages.

Due, in part, to a combination of mucociliary clearance and inherent high lung tissue permeability, achieving prolonged drug retention within the pulmonary tissue at a therapeutically acceptable concentration remains a major challenge.^7^ Several strategies have evolved including (i) the diffusion microkinetic theory—where a high membrane partitioning of lipophilic bases into phospholipid bilayers explains the long duration of action of some bronchodilators;^10^ (ii) receptor kinetics, in which slow receptor off-rates have been proposed as a hypothesis for the enhanced duration of action observed with both inhaled β_2_-agonists and muscarinic M3 receptor antagonists;^11^ and (iii) reduction in solubility, where the slow dissolution of drug particles into the lung-lining fluid affords the potential for extended lung retention.^1^ In addition, sustained-release formulations such as biodegradable polymer-based particles,^12,13^ liposomes,^14^ and poly(ethylene glycol)–drug ester conjugates^15^ have all been assessed to increase drug residence time within the lung tissue. Over recent years, strategies to reduce pulmonary absorption by modifying the physicochemical properties of the therapeutic compound through drug design have resulted. At the forefront of these approaches was the observation that dibasic compounds per se have a very high capacity to exhibit long lung retention.^16−20^ However, care needs to be applied in this strategy to ensure that there is sufficient local concentration of unbound drugs to have the required pharmacodynamic (PD) benefit. In addition, for intracellular targets, such as compounds designed to inhibit phosphatidylinositol 3-kinase (PI3K), a narrow physicochemical property window has been suggested for the balance of high lung tissue binding and cell permeation to enable the required long pharmacodynamics effect.^21^ Taking into account the requirement to balance extended lung retention with a sustained concentration of the free drug, we report here on our initial work to evaluate a new prodrug approach for extended lung tissue retention. Its concept is based on attaching a known poorly lung tissue-retentive compound to a lung tissue-retentive dibasic chemical substance through a pH-triggered release linker (slowly cleaved at pH > 6.5). The hypothesis is that the active drug would be slowly released in a controlled manner from the lung tissue depot, thus increasing the chances of successfully achieving “once-a-day” dosing regimens. Such a strategy could potentially be applied to inhaled drug classes acting on both cell surface and intracellular pharmacological targets as the active drug could freely be absorbed through cell membranes from its lung tissue depot after release to achieve the required extended duration of effect (Figure 1).

To achieve our objective of developing a platform-based technology for inhaled lung delivery, the prodrug would need to:

- Be readily synthesized with the concept applicable to a range of chemical classes
- Be of low molecular weight to avoid long-term accumulation in the lung
- Substantially increase the solubility of the parent drug to prevent local irritancy
- Preferentially be pH-dependently cleaved (more stable at the pH of lung fluid (pH 6.5) and cleaved at the pH of blood (pH 7.4)).
- Possess a nontoxic lung tissue-retentive moiety (“anchor”) that can be easily excreted after activation.

## Results and Discussion

**Figure 2:**
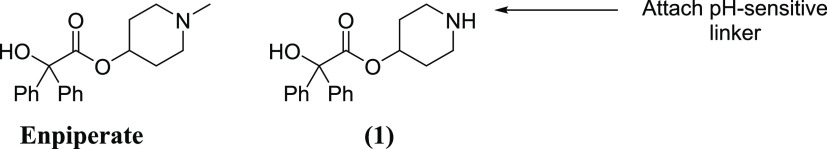
Chemical structure of enpiperate and the active metabolite **1** chosen for the feasibility study.

The prodrug design consisted of the parent drug bound to a lung-retentive moiety *via* a linker group, which bioactivates at a controlled rate to liberate the active drug molecule alongside nontoxic side products. To test the feasibility of this approach, we chose to functionalize the known muscarinic M_3_ receptor antagonist **1**, an active metabolite of enpiperate,^22−24^ as **1** possesses the required synthetic handle that can be readily modified with our recently disclosed pH-sensitive linking group (Figure 2).^25^

**Figure 3:**
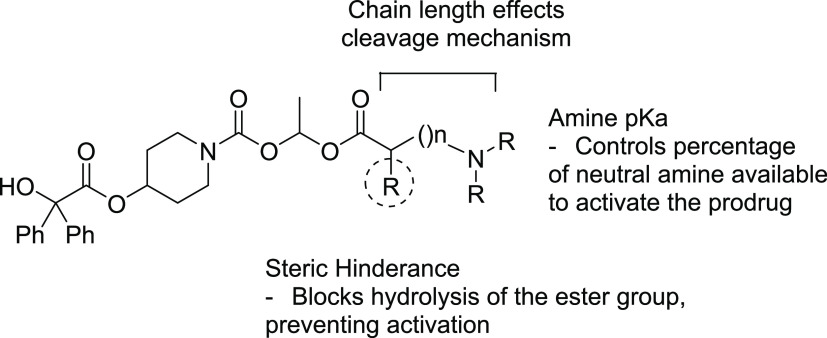
Potential rate-determining factors for hydrolysis of the prodrugs (**3**–**28**).

In our initial studies, we explored the synthesis of a series of monobasic amino acids attached to **1** via a carbamate linker to explore the aqueous pH stability (pH 6.5) on both the positioning of the amine, its basicity (p*K*_a_), and also the steric effect of substituents (Figure 3).

**Figure 4:**
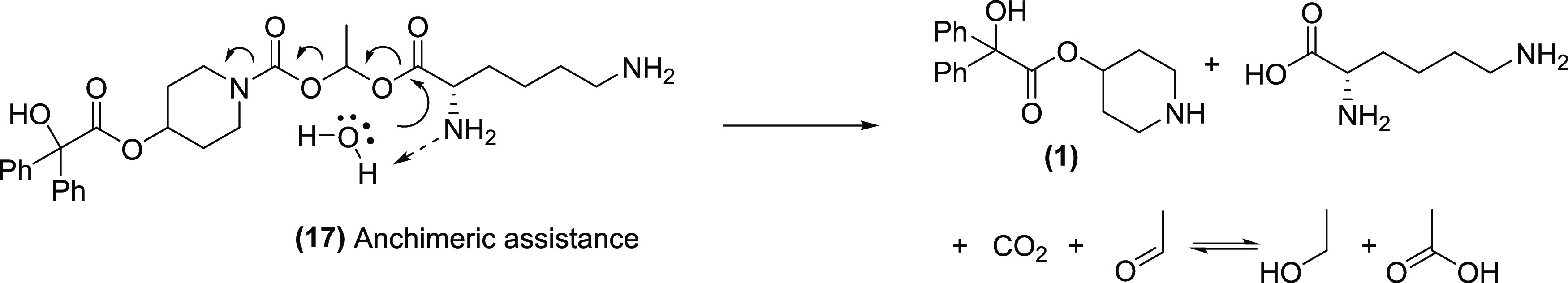
Proposed anchimeric-assisted cleavage mechanism shown for the arginine-based prodrug (**17**).

The p*K*_a_ of the amine governs the percentage ionized at the stated pH and is an important determinant in the hydrolytic cleavage of the carbamate by the anchimeric-assisted cleavage mechanism. The rate of cleavage would be pH-dependent as only the free amino compound could undertake the anchimeric-assisted delivery of water. NMR experiments in buffered pH 6.5 deuterated phosphate-buffered saline (PBS)/D_6_-DMSO demonstrated the clean conversion of **17** to **1**, arginine, and formaldehyde, which disproportionate to a mixture of acetic acid and ethanol (see Supporting Information S3) (Figure 4).^25,26^

The synthesized prodrugs were tested for their aqueous stability at pH 6.5 in an aqueous phosphate-buffered saline (PBS) (Table 1).

**Table 1:**
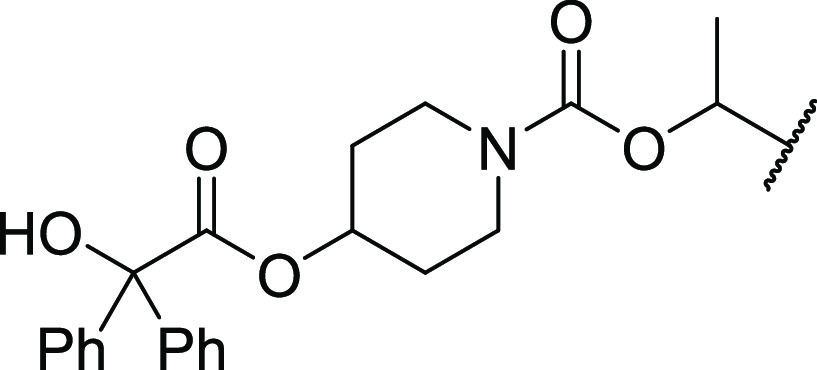

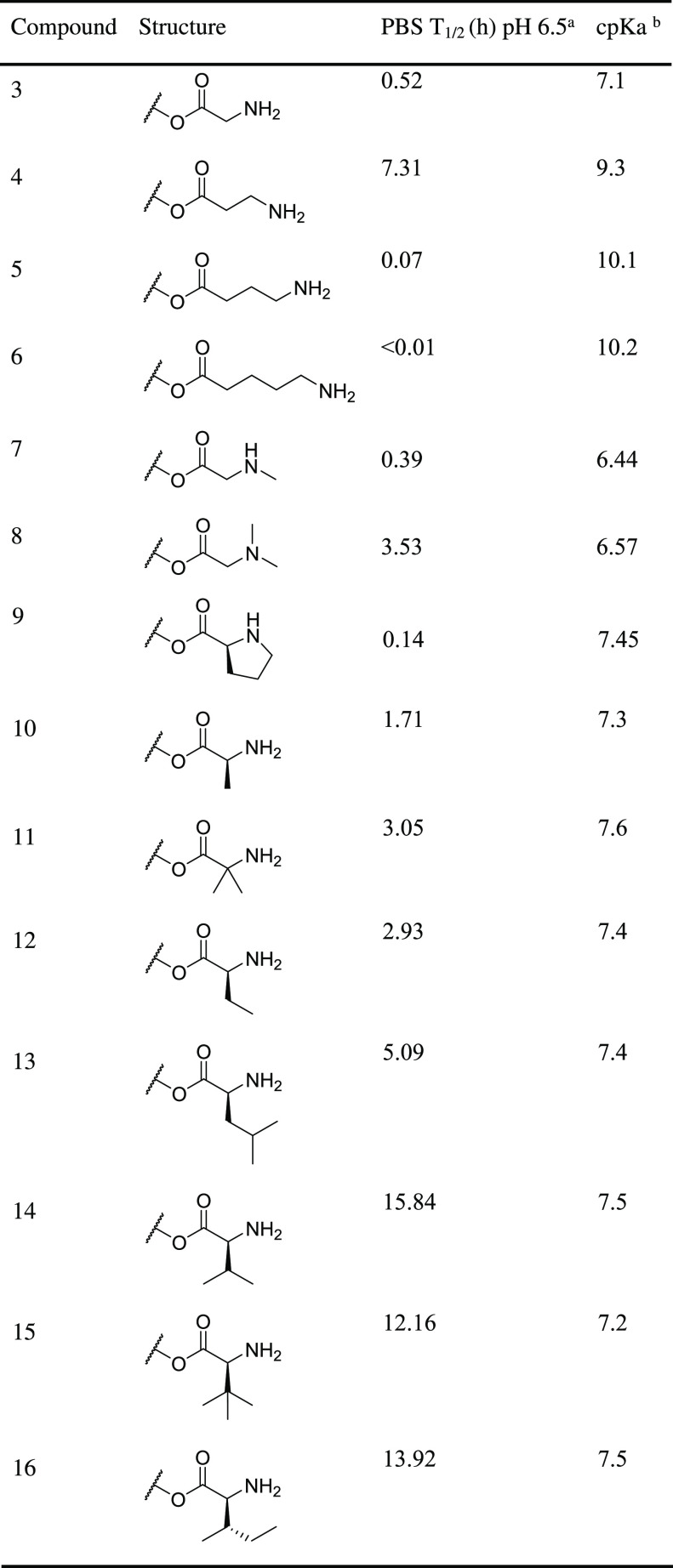
Aqueous Stability Tests at pH 6.5

a Prodrug stability was determined using pH 6.5 phosphate buffer solution as the reaction matrix. The decomposition of the deprotected prodrugs at 37 °C was monitored by liquid chromatography–mass spectrometry (LC–MS) to determine the half-life of the prodrug. Cleavage was stalled by the addition of acetic acid to the LC–MS sample, and the sample was frozen until characterization. Results show an average of *n* = 2 experiments.

b Calculator plugins were used for structure–property prediction and calculation, Marvin 18.30.0, 2018, ChemAxon (http://www.chemaxon.com).

The first parameter to be explored featured the position of the amine and how this might affect the rate of hydrolysis and the mechanism by which the prodrug cleaves. In the initial experiment, the glycine-derived prodrug **3** was shown to be readily hydrolyzed (*T*_1/2_ = 0.52 h), whereas the β alanine-derived prodrug **4** had increased stability (*T*_1/2_ = 7.3 h). This trend in stability was not continued in the γ- and δ-substituted prodrugs (**5** and **6**, respectively), indicating a change in hydrolysis mechanism from anchimeric-assisted hydrolysis to an intramolecular nucleophilic cleavage mechanism, subsequently proven by NMR that showed the appearance of pyrrolidin-2-one and piperidin-2-one, respectively (n.b. no azetid-2-one was observed in the cleavage of **4**). As expected, direct mono *N*-alkylation (**7**) reduced the p*K*_a_ of the amine, which slightly increased the cleavage rate (compared to **3**). Dialkylation (**8**), while again reducing the p*K*_a_, leads to a slower cleavage rate presumably for increased steric reasons and loss of H-bond donor properties. The use of proline (**9**) caused the cleavage rate to increase rapidly despite increasing the p*K*_a_; this was rationalized due to Thorpe–Ingold effects, which push the two reactive components together to relieve steric strain.^27,28^ The size of the amino acid side chain is another method by which the rate of prodrug hydrolysis can be controlled. By placing a large sterically hindering group next to the carbonyl group, an attack at this carbon would be sterically blocked and thus it was important to investigate how the size of the C-1 side chain affects its potential to sterically hinder hydrolysis. This data demonstrated how bulkier amino acid side chains slowed the hydrolysis of the prodrug by sterically hindering the anchimeric-assisted delivery of water. This resulted in slower cleavage rates in prodrugs (**12**–**16**) compared to that in **3**.

From the monobasic prodrug cleavage data, it was clear that there were two possible approaches when designing dibasic amino acids. One route was to include an α-positioned amino acid with a large bulky side group to reduce the rate of cleavage, while the second approach involved a β-positioned amino acid, which would rely on the higher amine p*K*_a_ to reduce the cleavage rate. Once incorporated, these α- and β-amino groups could then be derivatized to contain a second basic component, in which the cleavage mechanism would rely on anchimeric assistance (Table 2).

**Table 2:**
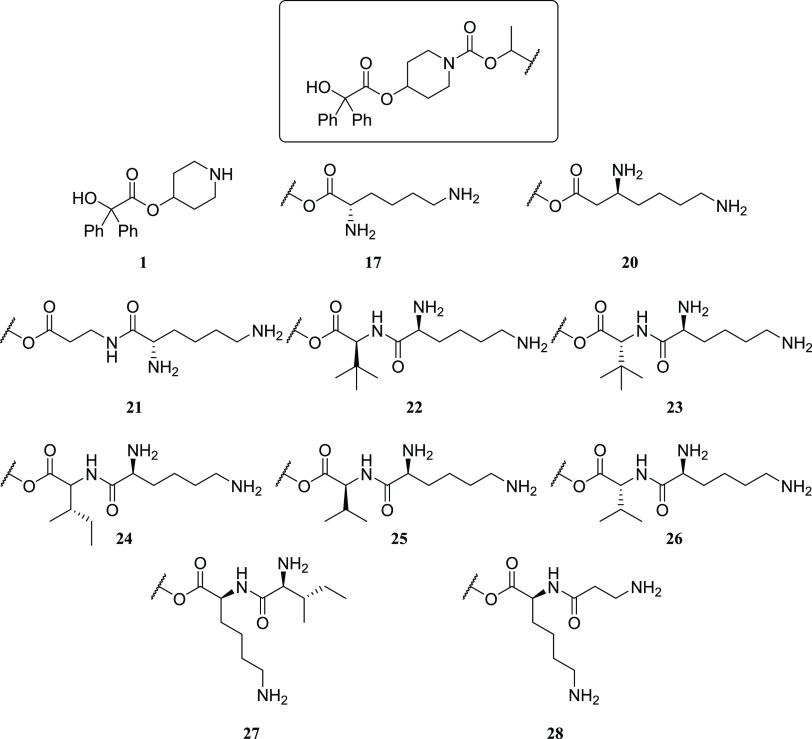
Exploration of Dibasic Prodrug Stability in pH 6.5 and 7.4 PBS, Rat Lung Homogenate, Rat Lung Tissue Binding, and Blood Stability^b^^,^^g^

example	PBS *T*_1/2_ (h) pH 6.5^a^	PBS *T*_1/2_ (h) pH 7.4^a^	cp*K*_a_^1^	cp*K*_a_^2^	rat lung homogenate *T*_1/2_ (h) pH 6.8^c^	rat blood *T*_1/2_ (h) pH 7.2^d^	rat lung homogenate binding (% free)^e^
1			10		stable	stable	18.3 ± 1.70
17	3.90 ± 0.05	1.10 ± 0.02	7.4	10.1	f	f	f
20	29.10 ± 1.49	5.90 ± 0.13	9.4	10.2	3.1 ± 0.38	0.3 ± 0.02	f
21	54.50 ± 3.16	44.8 ± 0.88	8.4	10.1	0.6 ± 0.04	f	f
22	186.00 ± 0.79	67.00 ± 0.23	8.4	10.1	2.5 ± 0.07	0.1 ± 0.01	f
23	58.60 ± 4.91	36.6 ± 1.70	8.4	10.1	25.4 ± 1.94	7.0 ± 0.82	0.50 ± 0.10
24	17.6 ± 1.26	8.70 ± 0.69	8.4	10.1	0.8 ± 0.08	f	f
25	14.4 ± 1.68	4.40 ± 0.39	8.4	10.1	0.6 ± 0.09	f	f
26	8.70 ± 1.06	1.80 ± 0.20	8.4	10.1	3.4 ± 0.08	0.6 ± 0.07	f
27	4.90 ± 0.06	0.90 ± 0.04	10.1	8.5	f	f	f
28	102.20 ± 3.86	11.50 ± 0.73	10.1	9.00	0.7 ± 0.21	f	f

a Buffer stability was determined in triplicate at 37 °C using pH 6.5 or 7.4 phosphate buffer solution as the reaction matrix.

b Calculator plugins were used for structure–property prediction and calculation, Marvin 18.30.0, 2018, ChemAxon (http://www.chemaxon.com).

c Rat lung homogenate stability was determined in triplicate at 37 °C, using a 1 in 4 aqueous dilution of rat lung homogenate as the reaction matrix. Species: Crl Wistar Han, male.

d Rat blood stability was determined in triplicate at 37 °C using rat blood as the reaction matrix.

e Rat lung homogenate. Binding was determined in triplicate at 37 °C, using a 1 in 4 aqueous dilution of rat lung homogenate as the reaction matrix.

f Not determined as the previous biological stability was unacceptable.

g For procedures (a), (c), (d), and (e), the mean determined value is displayed ± standard deviation. Quantified using mass-spec analysis against internal standards labetalol and reserpine.

**Figure 5:**
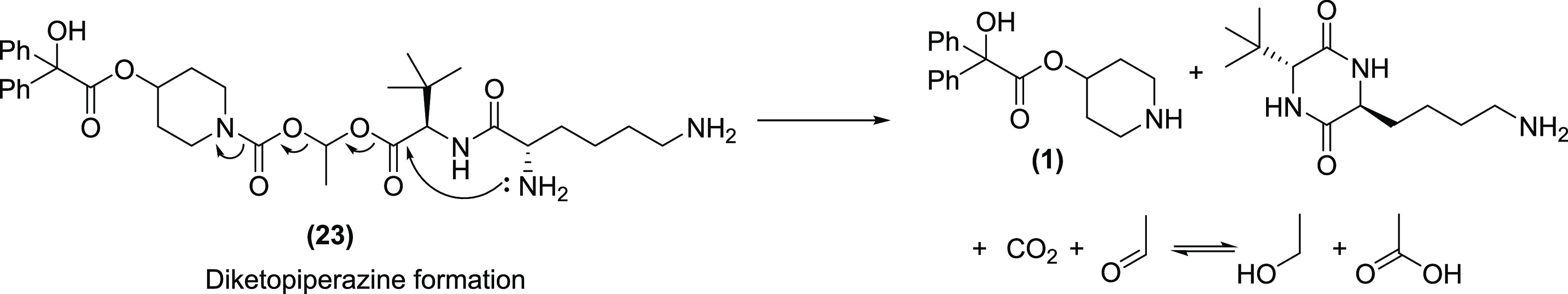
Proposed diketopiperazine cleavage mechanism.

The PBS buffer stability was determined to provide the maximum possible stability the prodrugs would have in the complete absence of enzymatic activity, i.e., the cleavage rate due to self-activation. This was tested in triplicate at 37 °C at both pH 6.5 and 7.4. In all results, a clear propensity for cleavage at higher pH was observed, demonstrating the pH-sensitive nature of the linking group. Compounds **17** and **20** displayed the same trend as was witnessed for the monobasic prodrugs, with the α-amino group giving a much faster cleavage rate than the β-amino prodrug. Unfortunately, as the mono-amino acid prodrugs (3–16) did not have a sufficient stability profile to progress further, an alternative strategy was sourced. To incorporate a sterically hindered α-positioned amino group, a series of dibasic dipeptides were synthesized. The sterically hindered ester group would then have increased resilience to nucleophilic attack, and thus the prodrug cleavage would depend less on the terminal amine p*K*_a_. This is because cleavage would now take place via an intramolecular diketopiperazine (DKP) formation mechanism, in which the intramolecular cyclization would occur via the nucleophilic attack of the primary amine of the first amino acid to the ester. This would create a 6-membered diketopiperazine, the formation of which would be more heavily influenced by steric hindrance than by the p*K*_a_ of the nucleophilic amine. The rate of cleavage would be pH-dependent as only the free amino compound **23** could undertake the cyclization to the diketopiperazine. NMR experiments in buffered pH 6.5 deuterated PBS/D_6_-DMSO demonstrated the clean conversion to **1**, substituted diketopiperazine, and formaldehyde, which disproportionate to a mixture of acetic acid and ethanol (see Supporting Information S2) (Figure 5).

**Figure 6:**
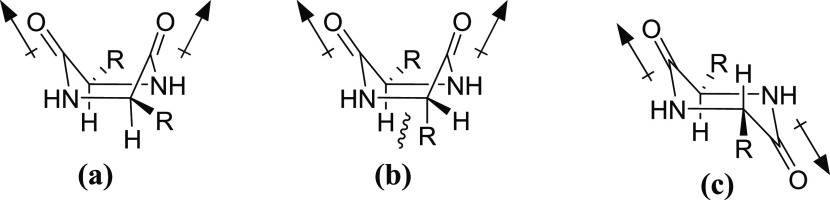
Dipeptide low-energy conformation: (a) boat conformation with natural amino acid isomers, (b) boat conformation with unnatural amino acid isomers, and (c) chair conformation with balanced dipoles.

The prodrugs (**21**–**28**) were found to have a range of stabilities in phosphate buffer. As previously witnessed, the bulkier the C-1 side chain the lower the rate of cleavage, and hence the rate increases from compound **27**, which has a relatively small α-substituent, to the highly hindered tertiary butyl group in compound **22**. The most interesting results came from **23** and **26**. To increase the chemical stability and reduce internal steric clashes, diketopiperazines place both groups in pseudo-equatorial positions resulting in a boat shape configuration (Figure 6a).^29,30^ For this reason, it was hypothesized that by inverting the stereochemistry of one of the amino acids in the dipeptide, a destabilizing steric clash would be formed, which would make the DKP less likely to form (Figure 6b), thus increasing the stability of the prodrug. However, when the stability of compounds **23** and **26** was tested, the prodrug stability in aqueous buffer did not improve relative to compounds **22** and **25**. This could be explained by a shift in the DKP structure to a more chair-like conformation to reduce the steric clash between the hydrogen atom and the side chain and to balance the two carbonyl dipoles, which would be uneven in the boat conformation for the l,l-dipeptide (Figure 6c).

**Figure 7:**
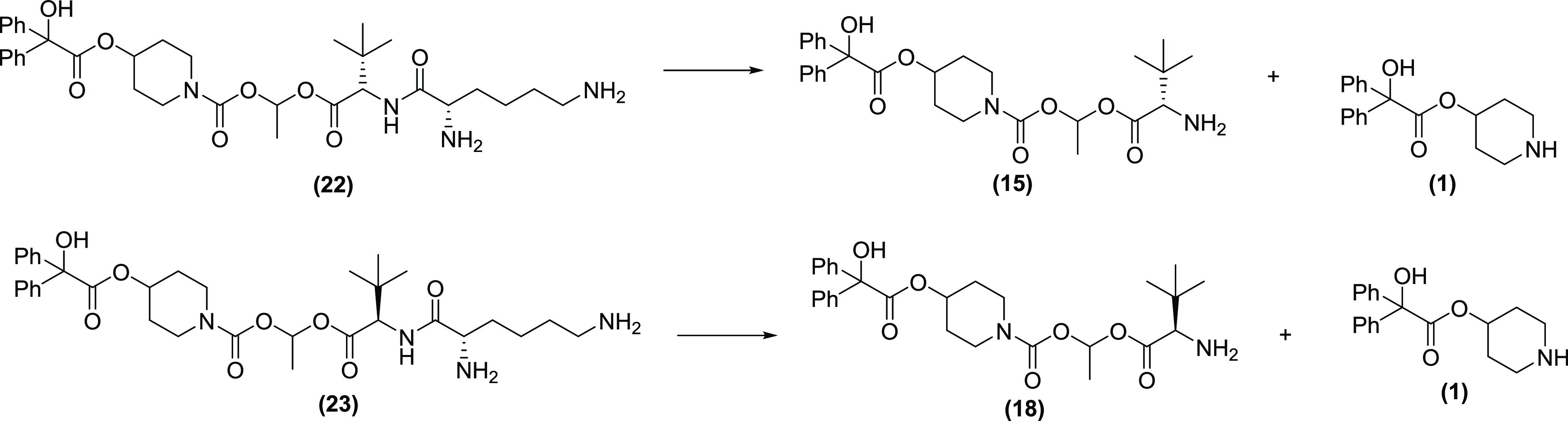
Proposed potential peptide cleavage pathway giving intermediates **15** and **18** along with released drug **1**.

Based on the phosphate stability studies and the prediction that enzymatic activity will increase the cleavage rate in biological media, the only compounds that proceeded to rat lung homogenate studies were compounds **20**, **21–26**, and **28**. Han Wistar rat lung homogenate was prepared, and the pH (at a dilution of 1–4 in water) was experimentally determined to be 6.8. This meant that should our prodrugs not exhibit any enzymatic cleavage, the rate of pure intramolecular cleavage should be faster than in phosphate buffer at pH 6.5 but slower than at pH 7.4. The results demonstrated that most of the prodrugs were indeed subjected to a high level of enzymatic metabolism. The other clear conclusion to be drawn from the lung homogenate stability results was that compounds **23** and **26**, in which the chirality of one of the amino acids was inverted from their natural isomer (**22** and **25**, respectively), were much more stable relative to their natural isomer matched pair. It is thought that by inverting the amino acid chirality, enzymatic cleavage at either the ester or amide bond will be reduced, possibly through the removal of the compound recognition for the enzymes’ active site. To understand the enzymatic cleavage for compounds **22** and **23**, the lung homogenate stability assay was repeated to detect the formation of the intermediate species **15, 18**, and **1** produced if the terminal lysine residue was removed due to peptide amide bond hydrolysis (Figure 7).

**Figure 8:**
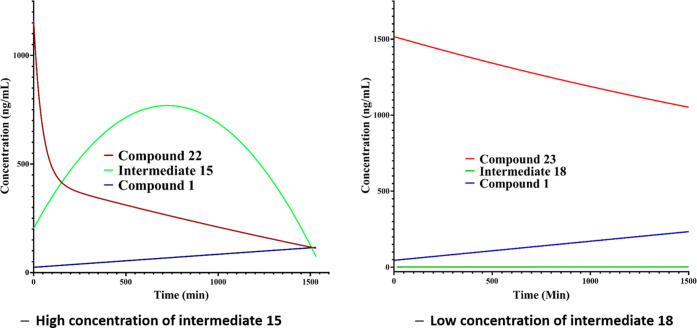
Analysis of the route of cleavage for compounds **22** and **23** in rat lung homogenate, demonstrating different hydrolysis routes. The left panel demonstrates the enzymatic cleavage of the natural isomer of prodrug **22** (red) into the monobasic intermediate **15** (green) followed by further chemical cleavage into the parent drug **1** (blue). The right panel demonstrates no enzymatic cleavage of the unnatural amino acid prodrug **23** (red), as indicated by the lack of detection of intermediate **18** (green). Instead, only the chemical cleavage of **23** into parent drug **1** (blue) is detected. Compounds were incubated into rat lung homogenate at a nominal concentration of 1500 ng/mL and breakdown followed over 1500 min using MS/MS analysis against authenticated samples. Lower limits of quantification (LLOQs): **1** (20 ng/mL), **22** (10 ng/mL), **15** (0.2 ng/mL), **23** (2 ng/mL), and **18** (1 ng/mL). Data processed using GraphPad PRISM V7.02.

The results proved quite revealing with the natural amino acid analogue **22**, clearly showing the intermediate **15**, whereas **23** showed no evidence of intermediate **18**, demonstrating that prodrug cleavage was occurring predominantly by a hydrolysis mechanism (Figure 8).

Interestingly for **23**, the rat lung homogenate stability and PBS stability were comparable (*T*_1/2_ 25.4 and 36.6 h, respectively), giving further evidence that the cleavage of **23** was mainly occurring through a nonenzymatic hydrolysis mechanism. Due to the chemical stability observed in rat lung homogenate for **23**, it was possible to measure its rat lung homogenate binding. Compound **23** was 0.5% unbound compared to compound **1**, which showed an unbound fraction of 18.3%. In addition, **23** had reasonable blood stability (*T*_1/2_ 7 h) compared to **22** (*T*_1/2_ 0.2 h). As a consequence of the encouraging *in vitro* data, the *in vivo* lung tissue retention capacity of **23** was evaluated in a rat intratracheal dosing pharmacokinetic (ITPK) study, where concentrations of compounds **23** and **1** would be measured in both rat lung and plasma after the intratracheal delivery of **23**.

When designing the pharmacokinetic study, it was important to ensure that the dosing concentration was high enough to allow for the detection of drug **1** and prodrug **23** at the final time point of 24 h. For this reason, the compounds were dosed at a relatively high concentration of 0.2 mg/kg and it was pleasing to note that **23** appeared as a clear solution in 5% EtOH in 95% PBS at pH 6.5. This demonstrates an immediate advantage of the dibasic prodrug system as drug insolubility can cause inflammation of the airway. After the parent muscarinic M3 receptor antagonist (**1**) was dosed via intratracheal administration (IT), blood samples were taken at 0.5, 1, and 3 h time points, and the concentration of drug was measured. In addition, the residual total lung concentration at 3 h was measured (Table 3).

**Table 3:** Plasma and Total Lung Levels Obtained Following IT Administration of **1**^a^

compound **1** plasma concentration (ng/mL)	
time	(h) mean
0.5	<LLOQ
1	<LLOQ
3	<LLOQ
compound **1** total lung concentration (ng/mL)	
3	137 ± 31

a Compound **1** was dosed at 0.2 mg/kg IT in male rats (species: Crl Sprague Dawley, male, *n* = 3 per time point). Formulation of 5% ethanol in pH 6.5 PBS. <LLOQ = <lower limit of quantification, LLOQ = 25.0 ng/mL. The results shown are the mean of three replicates with standard deviation. Sample data analysis was performed at Sygnature Discovery, and the in-life phase was performed at Saretius Ltd.

Compound **1** could not be detected in blood as its plasma concentrations fell below the LLOQ for all time points. This was quite surprising as we know that drug **1** has reasonable plasma stability and so we might postulate that a combination of hepatic and extrahepatic clearance mechanisms might be involved, as it is known that basic and dibasic compounds are susceptible to organo cation transporters (OCTs)^31^ and it could be that the compounds are rapidly excreted into urine, as this is the case for some of the inhaled muscarinic antagonists as OCTs are expressed in the kidneys. At 3 h, the percentage of **1** remaining in the lung was calculated as 0.33% of the initial calculated total dose administered. This result demonstrates the very poor lung retention of compound **1**, which would most likely be translated into a very short observed duration of action.

For the ITPK study of compound **23**, plasma samples were then taken at 0.25, 0.5, 1, 2, 3, 5, 8, and 24 h time points, and the concentration of **1** and **23** was measured. At 24 h, the terminal total lung concentrations of **1** and **23** were measured (Table 4).

**Table 4:** Plasma and Total Lung Levels Obtained Following IT Administration of **23**^a^

time (h)	rat plasma concentration **1** (ng/mL)	rat plasma concentration **23** (ng/mL)	rat total lung concentration **1** (ng/mL)	rat total lung concentration **23** (ng/mL)
0.25	<LLOQ	2.03 ± 0.45		
0.5	<LLOQ	1.63 ± 0.14		
1	<LLOQ	2.10 ± 0.05		
2	<LLOQ	1.25 ± 0.10		
3	<LLOQ	<LLOQ		
5	<LLOQ	<LLOQ		
8	<LLOQ	<LLOQ		
24	<LLOQ	<LLOQ	361 ± 32	20,000 ± 611

a Compound **23** was dosed at 0.2 mg/kg IT in a male rat (species: Crl:Sprague Dawley, male, *n* = 3 per time point). Formulation of 5% ethanol in pH 6.5 PBS. <LLOQ = <lower limit of quantification, LLOQ = 1.0 ng/mL (**23**) 20.0 ng/mL (**1**). The results shown are the mean of three replicates with standard deviation. Sample data analysis was performed at Sygnature Discovery, and the in-life phase was performed at Saretius Ltd.

As in the first study, compound **1** was not detected in any plasma samples and only a very low concentration of **23** was detected in the plasma up to 2 h post dosing. However, at 24 h, the total rat lung concentration was measured at 20,000 ng/mL (31.2 μM) for **23** (54% of the total dose delivered based on the calculated mass balance from the amount recovered in the lung tissue as a fraction of the total drug administered, assuming 100% lung deposition) and 361 ng/mL for the released active drug **1**, which equates to an observed total lung concentration of 1.16 μM (an ∼27:1 ratio of **23** to **1** (3.7 ± 0.1%)). When working with prodrugs, much care is required in the interpretation of pharmacokinetic results as the prodrug could break down to release the parent drug during sample preparation. This is unlikely to be the case in this study as **23** has a half-life of 25.4 h when incubated at 37 °C in the presence of rat lung homogenate, while sample preparation requires 10 min at an ambient temperature. However, to alleviate concerns around the prodrug stability in lung samples during sample preparation, a set of control experiments were conducted, where **23** in the IT dosing vehicle was spiked into rat lungs followed by their homogenization or directly into rat lung homogenates before samples were prepared for mass spectral quantification. There was very little evidence for the release of **1** from prodrug **23** during the sample workup. The initial fraction of **1** in the **23** stock solution was determined as ∼0.3–0.4%, whereas after sample preparation from lung homogenate or spiking into lungs, the percentage of **1** was quantified as 0.62 ± 0.03% (*n* = 3) and 0.56 ± 0.02% (*n* = 3), respectively. This would suggest that the conversion of **23** into **1** occurred within the lung tissue during the time course of the ITPK study and not during analytical sample preparation.

## Synthesis

**Scheme 1:**
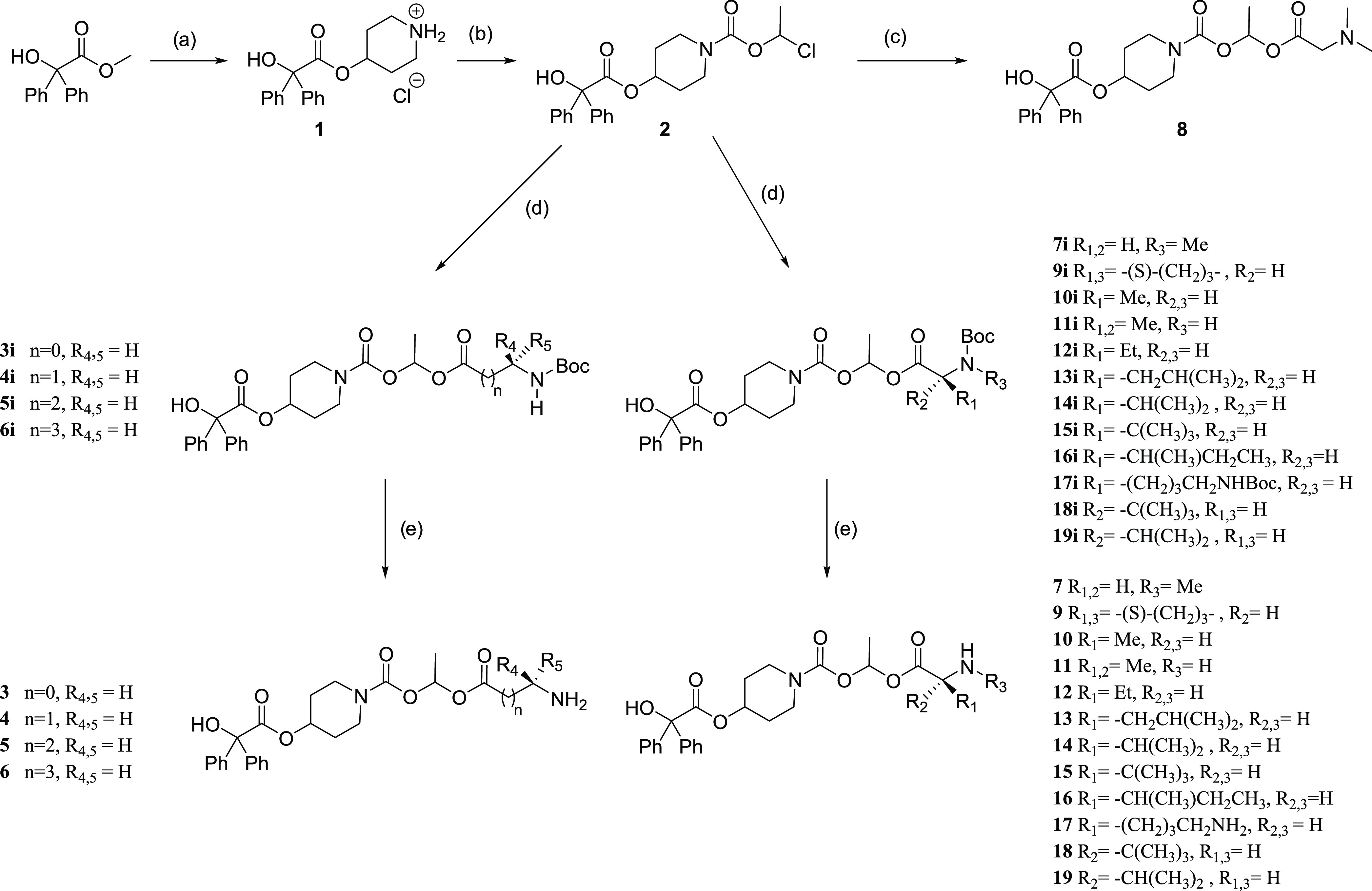
Prodrugs (**3–19**) Synthesized through a Common Synthetic Strategy (a) (i) Methyl benzilate (1 equiv), *tert*-butyl 4-hydroxy piperidine-1-carboxylate (0.75 equiv), sodium (0.075 equiv), and triethylamine (5 equiv) in toluene 60 °C, 16 h, 17%. (ii) 4N HCl in 1,4-dioxane, room temperature (rt) 18 h, 100%. (b) **1** (1 equiv), 4-methylmorpholine (3.3 equiv), 1-chloroethyl chloroformate (1.1 equiv) in dry dichloromethane (DCM), −10 °C to rt, 71%. (c) *N*,*N*-Dimethylglycine (1.2 equiv), silver(I) oxide (1.2 equiv), Bu_4_N^+^Br^–^ (0.2 equiv), toluene, 65 °C, 6 h, 43%. (d) *N*-Boc amino acids (1.2 equiv), silver(I) oxide (1.2 equiv), Bu_4_N^+^Br^–^ (0.2 equiv), toluene, 65 °C, 6 h, 21–85%. (e) (i) Trifluoroacetic acid (TFA) in CH_2_Cl_2_, 2 h, rt evaporate. (ii) 2N HCl in diethyl ether (evaporate × 2), 100%, compounds isolated as either the mono- or dihydrochloride salts.

The prodrugs (**3–19**) were synthesized through a common synthetic strategy (Scheme 1). Methyl benzilate was transesterified using a catalytic amount of sodium and *N*-Boc-4-hydroxy piperidine to give after Boc-deprotection **1** isolated as the stable HCl salt.^32^ Subsequent reaction of **1** with 1-(chloromethoxy)ethyl carbonochloridate affords the common precursor **2**, which can be coupled with Boc-protected amino acids using silver(I) oxide and tetra-*N*-butylammonium bromide in toluene at 50 °C^25^ to give the Boc-protected compounds (**3i–7i**, **9i–18i**), which were deprotected using anhydrous HCl in 1,4-dioxane to give (**3–19**) isolated as their mono- or dihydrochloride salts.

**Scheme 2:**
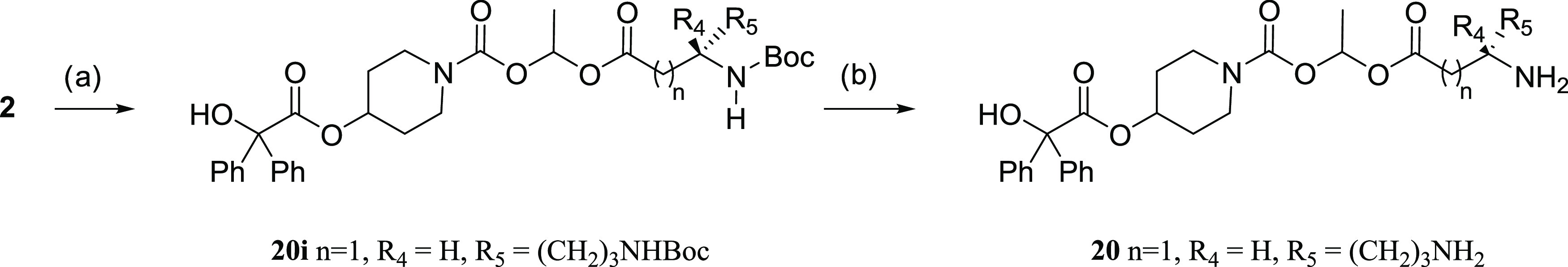
Crude Product Protected with Boc Anhydride to Give **20i** and Deprotected with Anhydrous HCl in 1,4-Dioxane to Give **20** as the Stable di-HCl Salt (a) (i) Methyl benzilate (1 equiv), *tert*-butyl 4-hydroxy piperidine-1-carboxylate (0.75 equiv), sodium (0.075 equiv), and triethylamine (5 equiv) in toluene 60 °C, 16 h, 17%. (ii) 4N HCl in 1,4-dioxane, room temperature (rt) 18 h, 100%. (b) 1 (1 equiv), 4-methylmorpholine (3.3 equiv), 1-chloroethyl chloroformate (1.1 equiv) in dry dichloromethane (DCM), −10 °C to rt, 71%. (c) *N*,*N*-Dimethylglycine (1.2 equiv), silver(I) oxide (1.2 equiv), Bu4N^+^Br^–^ (0.2 equiv), toluene, 65 °C, 6 h, 43%. (d) *N*-Boc amino acids (1.2 equiv), silver(I) oxide (1.2 equiv), Bu4N^+^Br^–^ (0.2 equiv), toluene, 65 °C, 6 h, 21–85%. (e) (i) Trifluoroacetic acid (TFA) in CH_2_Cl_2_, 2 h, rt evaporate. (ii) 2N HCl in diethyl ether (evaporate × 2), 100%, compounds isolated as either the mono- or dihydrochloride salts.

In the synthesis of compound **20**, a similar route was applied, reacting **2** with (*S*)-7-(((benzyloxy)carbonyl)amino)-3-((*tert*-butoxycarbonyl)amino)heptanoic acid under the standard conditions. Hydrogenation afforded **20**; however, partial decomposition was observed, so the crude product was protected with Boc anhydride to give **20i** and deprotected with anhydrous HCl in 1,4-dioxane to give **20** as the stable di-HCl salt (Scheme 2).

**Scheme 3:**
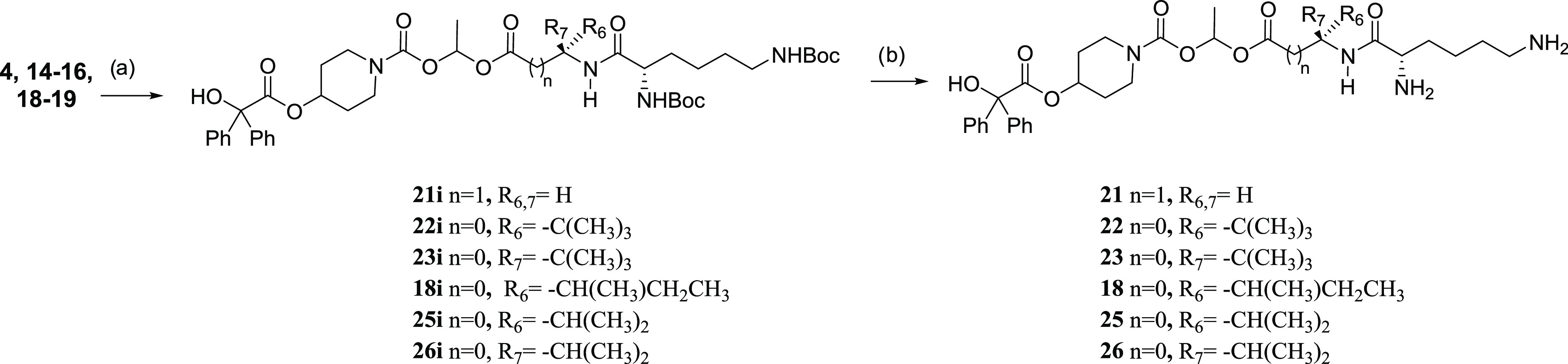
Amines (**4, 14–16**, and **18**–**19**) Were Reacted with Di-*tert*-butoxycarbonyl-lysine under Standard Peptide Coupling Conditions to Afford Compounds (**21i**–**26i**), Which Were Then Deprotected Using Anhydrous HCl in 1,4-Dioxane to Give (**21**–**26**) (a) Di-*tert*-butoxycarbonyl-lysine, 1-[bis(dimethylamino)methylene]-1*H*-1,2,3-triazolo[4,5-*b*]pyridinium 3-oxide (HATU) (1.5 equiv), 4-dimethylaminopyridine (DMAP) (0.5 equiv) and *N*,*N*-diisopropylethylamine (DIPEA) (6 equiv), CH_2_Cl_2_, rt, 6–20 h, 37–95%. (b) (i) TFA in CH_2_Cl_2_, 2 h, rt evaporate and (ii) 2N HCl in diethyl ether (evaporate × 2), 100%, compounds isolated as the dihydrochloride salt.

For the synthesis of compounds (**21**–**26**), the amines (**4, 14–16**, and **18**–**19**) were reacted with di-*tert*-butoxycarbonyl-lysine under standard peptide coupling conditions to afford compounds (**21i**–**26i**), which were then deprotected using anhydrous HCl in 1,4-dioxane to give (**21**–**26**) isolated as their dihydrochloride salts. The compounds were used without further purification (Scheme 3).

**Scheme 4:**
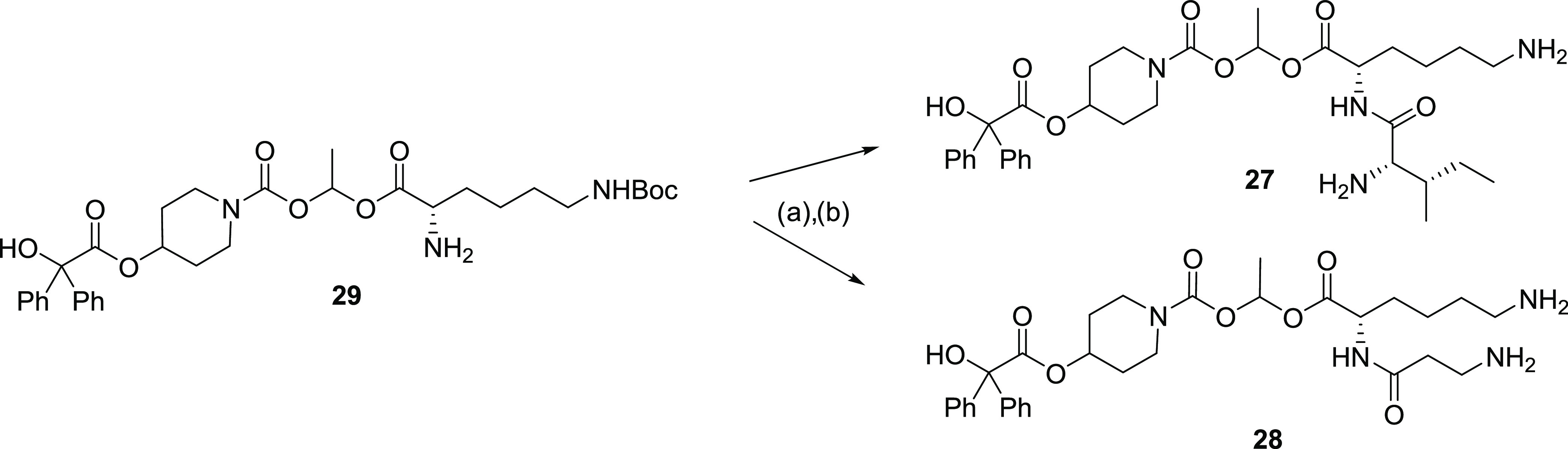
Compounds **27** and **28** Were Synthesized by Reacting **29** with (*tert*-Butoxycarbonyl)-l-isoleucine and 3-((*tert*-Butoxycarbonyl)amino)propanoic Acids, Respectively (a) (*tert*-Butoxycarbonyl)-l-isoleucine (**27i**, 44%), 3-((*tert*-butoxycarbonyl)amino)propanoic (**28i**, 39%), HATU (1.5 equiv), DMAP (0.5 equiv) and DIPEA (6 equiv), CH_2_Cl_2_, rt, 6–20 h, 37–95%. (b) (i) TFA in CH_2_Cl_2_, 2 h, rt evaporate and (ii) 2N HCl in diethyl ether (evaporate × 2), 100%, compounds isolated as the dihydrochloride salt.

Finally, compounds **27** and **28** were synthesized by reacting **29** with (*tert*-butoxycarbonyl)-l-isoleucine and 3-((*tert*-butoxycarbonyl)amino)propanoic acids, respectively, under standard peptide coupling conditions to give **27** and **28** after deprotection (Scheme 4^4^.).

## Conclusions

From consideration of the observation that dibasic compounds *per se* possess very good pharmacokinetic lung retention, a series of monobasic and dibasic prodrugs were synthesized and evaluated for their lung tissue binding and stability. Compound **23** was highlighted as a dibasic prodrug with the correct balance of measured lung tissue binding and chemical instability in PBS. The further evaluation suggested that the breakdown of prodrug **23** to active drug **1** occurred mainly through a pH-dependent diketopiperizine-forming cascade hydrolysis mechanism. The resulting prodrug **23** demonstrated high aqueous solubility and was dosed in a rat ITPK study to determine its pharmacokinetic profile. Quantification of plasma levels demonstrated very little systemic plasma exposure of **23** and released active drug **1** (**1** was not detected at any time points). However, at 24 h post dose, a high total lung concentration of **23** was observed along with the released active drug **1**. These initial results demonstrate a substantial increase in the lung residency of **1**, when administered as the prodrug conjugate **23**, when compared to dosing **1** alone (0.33% of a total administered dose observed at 3 h.). This result correlates with the observed increase in rat homogenate lung tissue binding between **1** (18.3% free) and **23** (0.5% free). The *in vivo* data supports an intrinsic pharmacokinetic benefit (by increasing concentrations of the active drug **1** and, more importantly, creating a reservoir of prodrug **23**, potentially slow-releasing further active drug **1**). It is our hypothesis that there should be an extended PD effect observed, as long as there is a sufficient local concentration of active drug **1** present. We appreciate that the safety implications of the extensive lung retention of **23** have not been fully investigated and that a fine-tuning to achieve a more desirable balance between lung tissue retention and duration of pharmacological action might be required. However, the attractiveness of the approach, in our opinion, lies precisely in the opportunity to select a prodrug with a tunable hydrolysis rate to achieve the required balance of prodrug lung retention and released active drug. Further work is ongoing within our laboratories to understand the lung tissue retention mechanisms of the dibasic prodrug moiety as well as further exploration of this new technology to other drug classes.

## Experimental Section

### Chemistry: General Methods

Chemicals and solvents were provided by Fisher Scientific U.K., Acros Organics, Sigma-Aldrich, Merck Millipore, or Fluorochem. All reactions were monitored by TLC using Merck Silica Gel 60 Å F254 TLC plates or by LC–MS. Unless otherwise stated, all compounds were dried under high vacuum either at rt or within an oven at 40 °C. LC–MS data was collected on a Shimadzu UFLCXR HPLC system coupled to an Applied Biosystems API 2000 LC/MS/MS electrospray ionization (ESI). The column used was a Phenomenex Gemini-NX 3 μm-110Â C18, 50 × 2 mm at 40 °C. The flow rate was 0.5 mL/min, and the UV detection was at 220 nm and 254 nm. Method 1 for the LC–MS ran for 1 min at 5% B; 5 to 98% B over 2 min, 98% B for 2 min, 98 to 5% B over 0.5 min, and then 5% for 1 min. Method 2 for the LC–MS ran for 1.5 min at 10% B; 10 to 98% B over 8 min; 98% B for 2 min; 98 to 10% B over 0.5 min, and then 10% B for 1 min, where solvent A is 0.1% formic acid in water and solvent B is acetonitrile. Unless otherwise stated, compounds reported had a purity >95% at the wavelength and method quoted. NMR spectroscopy was performed using a Bruker AV(III) HD 400 NMR spectrometer equipped with a 5 mm BBFO^+^ probe, recording ^1^H and ^13^C NMR at 400.25 MHz and 100.66 MHz, respectively, or a Bruker AV(III) 500 NMR spectrometer equipped with a 5 mm dual 1H/13C helium-cooled cryoprobe, recording ^1^H and ^13^C NMR at 500.13 MHz and 125.77 MHz, respectively. NMR data was processed using iNMR (version 5.5.7) referencing spectra to residual solvents. Chemical shifts are quoted as δ: values in ppm; coupling constants *J* = are given in Hz, and multiplicities are described as follows: s, singlet; d, doublet; t, triplet; q, quartet; qi, quintet; s, septet; m, multiplet; app, apparent; and bs, broad singlet.

Nonstandard abbreviations used in experimental: calculated (calcd), electrospray ionization (ESI), flash chromatography (FC), high-performance liquid chromatography (HPLC), high-resolution mass spectrometry (HRMS), liquid chromatography–mass spectrometry (LC–MS), preparative (PREP), reaction mixture (RM), reverse phase (RP), and thin-layer chromatography (TLC).

All compounds submitted for *in vitro* evaluation had a purity >95% and *in vivo* >99%.

#### General Chemistry Procedure **1**

To a solution of **2** (0.5 mmol, 1 equiv) in toluene (15 mL) were added the corresponding carboxylic acid (1.2 equiv), silver(I) oxide (1.2 equiv), and tetra-*n*-butylammonium bromide (0.2 equiv), and the reaction was heated at 65 °C between 6 and 8 h. The reaction was cooled, diluted with ethyl acetate (15 mL), filtered, and concentrated. The resulting residue was purified by chromatography on silica gel.

#### General Chemistry Procedure **2**

To a round-bottom flask was added the mono- or dihydrochloride salt as prepared in General Chemistry Procedure 1 or 3 (0.5 mmol, 1 equiv) in dry DCM (20 mL). To the suspension were added the corresponding protected amino acid (1.5 equiv), HATU (1.5 equiv), DMAP (0.5 equiv), and DIPEA (6 equiv). The resulting yellow solution was left to stir at room temperature for 6 h. The reaction was monitored by LC–MS, and once complete, the solution was diluted with DCM (50 mL) and washed with aqueous sodium hydrogen carbonate (3 × 25 mL). The organic layer was dried over anhydrous magnesium sulfate, filtered, and concentrated to give either the mono- or di-BOC-protected compounds, which were purified by chromatography to >99% purity.

#### General Chemistry Procedure **3**

To a round-bottomed flask containing the Boc-protected prodrug obtained (0.5 mmol, 1 equiv) in dry dichloromethane (5 mL) was added trifluoroacetic acid (1 mL). The reaction was stirred at room temperature for 3 h and was concentrated. A solution of 2N HCl in diethyl ether (3 mL) was added to the residue, and the mixture was stirred for 15 min and concentrated, azeotroping with dry toluene (2 mL). The residue was triturated with a further aliquot of 2 N HCl in diethyl ether and concentrated to afford either the mono- or dihydrochloride salt.

##### Piperidin-4-yl-2-hydroxy-2,2-diphenylacetate Hydrochloride **1**

Methyl benzilate (5.0 g, 20 mmol, 1 equiv) and t-butyl 4-hydroxy piperidine-1-carboxylate (3.02 g, 15 mmol, 0.75 equiv) were dissolved in hexane (60 mL) and stirred at room temperature. Sodium (0.034 g, 1.5 mmol, 0.075 equiv) and triethylamine (2.8 mL) were added. The reaction was then heated to 60 °C for 16 h, and the reaction solvent was evaporated. The crude mixture was then redissolved in 1:6 EtOAc/hexane and separated using silica gel column chromatography using a gradient of 1:6 to 1:4 EtOAc/hexane to obtain *tert*-butyl 4-(2-hydroxy-2,2-diphenylacetoxy)piperidine-1-carboxylate (1.467 g, 17%) as a colorless oil, which crystallized to a white solid on standing. ^1^H NMR (400 MHz; CDCl_3_) δ 7.31–7.48 (10H, m), 5.15 (1H, h, *J* = 4 Hz), 4.26 (1H, s), 3.31–3.35 (4H, m), 1.79–1.87 (2H, m), 1.59–1.68 (2H, m), 1.46 (9H, s). Calcd for C_24_H_29_NO_5_ = 411.50 found 412 [M + H]^+^. The intermediate from the above (0.801 g, 1.9 mmol, 1 equiv) was dissolved in DCM (10 mL) and 4N HCl in dioxane (1 mL) was added, and the reaction was stirred for 18 h during which time a white precipitate formed. Dry diethyl ether (50 mL) was added, and the white solid was filtered and washed with further portions of diethyl ether (2 × 20 mL) to obtain the title compound **1** (0.477 g, 71%) as a white solid. ^1^H (400 MHz, DMSO-*d*_6_) δ 8.87 (2H, bs), 7.40–7.26 (10H, m), 5.08 (1H, tt, *J* = 6.7, 3.3 Hz), 3.09–2.89 (4H, m), 2.06–1.93 (2H, m), 1.76 (2H, m). *m*/*z* (ESI; 98%) calcd for C_19_H_21_NO_3_ = 311.38 found 312.4 [M + H]^+^.

##### 1-Chloroethyl-4-(2-hydroxy-2,2-diphenylacetoxy)piperidine-1-carboxylate **2**

Compound **1** (0.1 g, 0.288 mmol, 1 equiv) was dissolved in dry DCM (10 mL) and cooled to −10 °C. 4-Methylmorpholine (0.105 mL, 0.951 mmol, 3.3 equiv) was added followed by 1-chloroethyl chloroformate (0.034 mL, 0.316 mmol, 1.1 equiv) dropwise over 1 min. The reaction was stirred for 2 h, and the solvents were removed under vacuum. The residue was dissolved in ethyl acetate (10 mL) and poured into 2N hydrochloric acid (10 mL). These organics were washed with water (2 × 10 mL), and then the organic layer was combined and dried over Na_2_SO_4_, filtered, concentrated under vacuum, and separated using silica gel column chromatography using a gradient of 1:4 EtOAc/petroleum ether (40:60) to obtain the title compound **2** as a colorless oil (0.477 g, 71%). ^1^H (400 MHz; CDCl_3_) δ 7.50–7.39 (4H, m), 7.41–7.31 (6H, m), 6.57 (1H, q, *J* = 5.8 Hz), 5.20 (1H, m), 4.23 (1H, s), 3.59–3.14 (4H, m), 1.95–1.82 (2H, m), 1.82 (3H, d, *J* = 5 Hz), 1.75–1.65 (2H, m). *m*/*z* (ESI; 97%) calcd for C_22_H_24_ClNO_5_ = 417.13 found 418.1 [M + H]^+^.

##### 1-(((*tert*-Butoxycarbonyl)glycyl)oxy)ethyl-4-(2-hydroxy-2,2-diphenylacetoxy)piperidine-1-carboxylate **3i**

The title compound was synthesized according to General Chemistry Procedure 1 using (*tert*-butoxycarbonyl)glycine and purified using silica gel column chromatography using a gradient of 1:5 EtOAc:(40–60) poly(ethylene terephthalate) (PET) to obtain the title compound **3i** (21%) as a colorless oil. ^1^H (400 MHz; CDCl_3_) δ 7.47–7.30 (10H, m), 6.83 (1H, q, *J* = 5 Hz), 5.21– 5.14 (2H, m), 3.40–3.80 (2H, m), 3.51–3.36 (2H, m), 3.35–3.20 (2H, m), 1.82–1.68 (5H, m), 1.49 (3H, d, *J* = 5 Hz), 1.45 (9H, s); ^13^C NMR (101 MHz, CDCl_3_) δ 173.8, 168.6, 155.6, 152.7, 141.8, 128.2, 128.2, 127.4, 90.5, 81.0, 80.1, 71.7, 42.3, 40.4, 29.9, 28.3, 19.8; *m*/*z* (ESI; 99%) calcd for C_29_H_36_N_2_O_9_ = 556.61 found 557.2 [M + H]^+^.

##### 1-(Glycyloxy)ethyl-4-(2-hydroxy-2,2-diphenylacetoxy)piperidine-1-carboxylate Hydrochloride **3**

The title compound was synthesized according to General Chemistry Procedure 3 from 3i to obtain the title compound **3** (quantitative) as a white solid. ^1^H (400 MHz; CDCl_3_) δ 8.10 (2H, bs) 7.46–7.30 (10H, m), 6.86 (1H, q, *J* = 4 Hz), 6.30 (2H, bs), 5.16 (1H, bs), 3.92 (2H, s), 3.51–3.15 (4H, m), 1.91–1.75 (2H, m), 1.74–1.57 (2H, m), 1.47 (3H, d, *J* = 4 Hz); ^13^C (101 MHz, D_6_-DMSO) δ 172.75, 166.63, 152.39, 143.77, 128.27, 127.95, 127.51, 90.94, 81.18, 70.36, 65.39, 40.61, 40.45 29.91, 19.98. *m*/*z* (ESI; 90%) calcd for C_24_H_28_N_2_O_7_ = 456.50 found 457.2 [M + H]^+^.

##### 1-((3-((*tert*-Butoxycarbonyl)amino)propanoyl)oxy)ethyl-4-(2-hydroxy-2,2-diphenylacetoxy)piperidine-1-carboxylate **4i**

The title compound was synthesized according to General Chemistry Procedure **1** using 3-((*tert*-butoxycarbonyl)amino)propanoic acid and purified using silica gel column chromatography using a gradient of 2:7 EtOAc:(40–60) PET to obtain **4i** (67%) as a colorless oil. ^1^H (400 MHz; CDCl_3_) δ 7.47–7.30 (10H, m), 6.77 (1H, q, *J* = 5 Hz), 5.22–5.09 (1H, m), 3.41–3.27 (6H, m), 2.51 (2H, t, *J* = 6 Hz), 1.93–1.75 (2H, m), 1.74–1.61 (2H, m), 1.48 (3H, d, *J* = 5 Hz), 1.44 (9H, s); ^13^C (101 MHz, CDCl_3_) δ 173.85, 170.67, 152.89, 141.80, 128.19, 128.16, 127.36, 90.10, 81.04, 79.36, 71.68, 40.45, 36.03, 34.73, 30.04, 29.80, 28.40, 19.80; *m*/*z* (ESI; 97%) calcd for C_30_H_38_N_2_O_9_ = 570.64 found 571.3 [M + H]^+^.

##### 1-((3-Aminopropanoyl)oxy)ethyl-4-(2-hydroxy-2,2-diphenylacetoxy)piperidine-1-carboxylate Hydrochloride **4**

The title compound was synthesized according to General Chemistry Procedure **3** from **4i** to obtain the title compound **4** (0.012 g, quantitative) as a white solid. ^1^H (400 MHz; D_6_-DMSO) δ 7.89 (2H, bs), 7.46–7.30 (10H, m) 6.80 (1H, q, *J* = 5 Hz), 5.84 (2H, bs), 5.18 (1H, s), 3.52–3.20 (6H, m), 2.94–2.82 (2H, m), 1.96–1.78 (2H, m), 1.75–1.57 (2H, m), 1.48 (3H, d, *J* = 5 Hz); ^13^C (101 MHz, D_6_-DMSO) δ 172.75, 169.20, 152.64, 143.77, 132.07, 129.14, 128.31, 128.26, 127.99, 127.94, 127.51, 127.41, 90.35, 81.17, 70.39, 38.55, 35.17, 34.74, 28.83, 15.64; *m*/*z* (ESI; 90%) calcd for C_25_H_30_N_2_O_7_ = 470.52 found 471.2 [M + H]^+^.

##### 1-((4-((*tert*-Butoxycarbonyl)amino)butanoyl)oxy)ethyl-4-(2-hydroxy-2,2-diphenylacetoxy)piperidine-1-carboxylate **5i**

The title compound was synthesized according to General Chemistry Procedure **1** using 4-((*tert*-butoxycarbonyl)amino)butanoic acid and purified using silica gel column chromatography using a gradient of 1:3 EtOAc/hexane to obtain the title compound **5i** (41%) as a colorless oil. ^1^H (400 MHz; CDCl_3_) δ 7.40 (10H, m), 6.78 (1H, q, *J* = 5 Hz), 5.17 (1H, m), 4.70 (1H, bs), 4.27 (1H, bs), 3.41–2.36 (8H, m), 1.81 (4H, m), 1.68 (2H, m), 1.48 (3H, d, *J* = 5 Hz), 1.45 (9H, s); ^13^C (101 MHz, CDCl3) δ 173.86, 171.34, 155.97, 152.86, 128.18, 128.16, 127.37, 90.08, 81.03, 71.75, 40.43, 39.67, 31.36, 30.02, 28.41, 25.00, 19.82; *m*/*z* (ESI; 97%) calcd for C_31_H_40_N_2_O_9_ = 584.67 found 585.2 [M + H]^+^.

##### 1-((4-Aminobutanoyl)oxy)ethyl-4-(2-hydroxy-2,2-diphenylacetoxy)piperidine-1-carboxylate Hydrochloride **5**

The title compound was synthesized according to General Chemistry Procedure **3** from **5i** to obtain the title compound **5** (quantitative) as a white solid. ^1^H (400 MHz; D_6_-DMSO) δ 7.60 (3H, bs), 7.45–7.3 (10H, m), 6.76 (1H, q, *J* = 5 Hz), 5.17 (1H, bs), 3.52–3.36 (2H, m), 3.33–3.23 (2H, m), 3.21–3.08 (2H, m), 2.56–2.46 (2H, m), 2.11–1.99 (2H, m), 1.92–1.75 (2H, m), 1.76–1.61 (2H, m), 1.48 (3H, d, *J* = 5 Hz); ^13^C (101 MHz, D_6_-DMSO) δ 174.18, 172.75, 152.70, 143.77, 128.31, 128.27, 128.00, 127.95, 127.51, 127.41, 90.06, 81.17, 67.92, 38.67, 38.41, 30.92, 30.73, 27.06, 22.96, 22.66, 20.07; *m*/*z* (ESI; 98%) calcd for C_26_H_32_N_2_O_7_ = 484.55 found 485.3 [M + H]^+^.

##### 1-((5-((*tert*-Butoxycarbonyl)amino)pentanoyl)oxy)ethyl-4-(2-hydroxy-2,2-diphenylacetoxy)piperidine-1-carboxylate Hydrochloride **6i**

The title compound was synthesized according to General Chemistry Procedure **1** using 5-((*tert*-butoxycarbonyl)amino)pentanoic acid and purified using silica gel column chromatography using a gradient of 1:3 EtOAc/hexane to obtain the title compound **6i** (85%) as a colorless oil. ^1^H (400 MHz; CDCl_3_) δ 7.50–7.30 (10H, m), 6.78 (1H, q, *J* = 5 Hz), 5.17 (1H, h, *J* = 4), 4.62 (1H, bs), 4.27 (1H, bs), 3.48–3.24 (4H, m), 3.12–3.06 (2H, m), 2.33 (2H, td *J* = 7 and 4 Hz), 1.90–1.79 (2H, m), 1.72–1.60 (4H, m), 1.56–1.49 (2H m), 1.48 (3H, d, *J* = 5 Hz), 1.45 (9H, s); ^13^C (101 MHz, CDCl_3_) δ 173.84, 171.49, 155.99, 152.84, 141.83, 128.15, 127.36, 89.98, 81.03, 79.15, 71.76, 53.44, 40.44, 40.05, 33.67, 30.04, 29.82, 29.70, 29.29, 28.42, 21.78, 19.85; *m*/*z* (ESI; 97%) calcd for C_32_H_42_N_2_O_9_ = 598.29 found 599.3 [M + H]^+^.

##### 1-((5-Aminopentanoyl)oxy)ethyl-4-(2-hydroxy-2,2-diphenylacetoxy)piperidine-1-carboxylate Hydrochloride **6**

The title compound was synthesized according to General Chemistry Procedure **3** from **6i** to obtain the title compound **6** (quantitative) as a white solid. ^1^H (400 MHz; D_6_-DMSO) δ 8.94 (1H, bs), 7.95 (3H, bs), 7.45–7.30 (10H, m), 6.77 (1H, q, *J* = 5 Hz), 5.12–5.01 (1H, m), 3.39–3.16 (4H, m), 3.08–2.91 (2H, m), 2.79 (2H, qt, *J* = 11.6, 5.8 Hz), 2.4 (1H, t, *J* = 7.5 Hz), 2.3 (1H, t, *J* = 7.3 Hz), 2.05–1.95 (1H, m), 1.83–1.72 (4H, m), 1.56–1.46 (1H, m), 1.4 (3H, d, *J* = 5.4 Hz); ^13^C (101 MHz, D_6_-DMSO) δ 174.63, 172.60, 158.55, 143.72, 129.18, 128.31, 128.26, 127.99, 127.94, 127.60, 127.51, 127.41, 81.17, 67.96, 65.39, 38.92, 38.77, 33.54, 27.03, 26.94, 26.67, 21.89; *m*/*z* (ESI; 98%) calcd for C_27_H_34_N_2_O_7_ = 498.58 found 499.3 [M + H]^+^.

##### 1-((*N*-(*tert*-Butoxycarbonyl)-*N*-methylglycyl)oxy)ethyl-4-(2-hydroxy-2,2-diphenylacetoxy)piperidine-1-carboxylate **7i**

The title compound was synthesized according to General Chemistry Procedure **1** using *N*-(*tert*-butoxycarbonyl)-*N*-methylglycine and purified using silica gel column chromatography using a gradient of 1:2 EtOAc:(40–60) PET to obtain **7i** (52%) as a colorless oil. ^1^H (400 MHz; CDCl_3_) δ 7.47–7.30 (10H, m), 6.82 (1H, q, *J* = 5 Hz), 5.20–5.12 (1H, m), 4.29 (1H, bs), 3.84 (2H, s), 3.50–3.17 (4H, m), 2.91 (3H, s), 1.82–1.65 (4H, m), 1.46 (12H, s); ^13^C (101 MHz, CDCl3) δ 173.85, 168.08, 141.81, 128.16, 127.36, 90.48, 81.03, 80.22, 71.65, 50.93, 50.08, 40.44, 35.50, 29.82, 28.32, 19.86; *m*/*z* (ESI; 97%) calcd for C_30_H_38_N_2_O_9_ = 570.64 found 571.3 [M + H]^+^.

##### 1-([Methylglycyl)oxy]ethyl-4-(2-hydroxy-2,2-diphenylacetoxy)piperidine-1-carboxylate Hydrochloride **7**

The title compound was synthesized according to General Chemistry Procedure **3** from **7i** to obtain the title compound **7** (quantitative) as a white solid. ^1^H (400 MHz; D_6_-DMSO) δ 9.36 (2H, bs), 7.44–7.32 (10H, m), 6.86 (1H, q, *J* = 5 Hz), 5.19 (1H, m), 4.29 (1H, bs), 3.88 (2H, s), 3.48–3.17 (4H, m), 2.84 (3H, s), 1.92–1.78 (2H, m), 1.76–1.62 (2H, m), 1.53 (3H, d, *J* = 5 Hz); ^13^C (101 MHz; D_6_-DMSO) δ 172.75, 172.6, 158.99, 143.77, 143.73, 129.79, 129.17, 129.13, 128.30, 128.26, 127.98, 127.94, 127.62, 127.51, 127.41, 90.99, 81.18, 70.35, 67.97, 48.66, 30.14, 19.92; *m*/*z* (ESI; 94%) calcd for C_25_H_30_N_2_O_7_ = 470.52 found 471.4 [M + H]^+^.

##### 1-((Dimethylglycyl)oxy)ethyl-4-(2-hydroxy-2,2-diphenylacetoxy)piperidine-1-carboxylate **8**

The title compound was synthesized according to General Chemistry Procedure **1** using dimethylglycine and purified by silica gel column chromatography using a gradient of 100% EtOAc to obtain the title compound **8** (43%) as a colorless oil. ^1^H (400 MHz; CDCl_3_) δ 7.46–7.30 (10H, m), 6.83 (1H, q, *J* = 5 Hz), 5.16 (1H, h, *J* = 4 Hz), 3.46–3.23 (4H, m), 3.20 (2H, s), 2.37 (6H, s), 1.89–1.87 (2H, m), 1.72–1.59 (2H, m), 1.50 (3H, d, *J* = 5 Hz); *m*/*z* (ESI; 97%) calcd for C_26_H_32_N_2_O_7_ = 484.22 found 485.3 [M + H]^+^.

##### 1-(*tert*-Butyl)-2-(1-((4-(2-hydroxy-2,2-diphenylacetoxy)piperidine-1-carbonyl)oxy)ethyl) (2*S*)-Pyrrolidine-1,2-dicarboxylate **9i**

The title compound was synthesized according to General Chemistry Procedure **1** using (*tert*-butoxycarbonyl)-l-proline and purified using silica gel column chromatography using a gradient of 1:2 EtOAc:(40–60) PET to obtain **9i** (67%) as a colorless oil. ^1^H (400 MHz; CDCl_3_) δ 7.40–7.29 (10H, m), 6.77 (1H, q, *J* = 5 Hz), 5.21–5.10 (1H, m), 4.29 (1H, bs), 3.59–3.27 (6H, m), 2.17–2.10 (1H, m), 2.01–1.82 (6H, m), 1.70–1.58 (2H, m), 1.51–1.47 (3H, m), 1.44 (9H, s); ^13^C DEPT (101 MHz; CDCl_3_) δ 128.12, 127.36, 90.53, 71.76, 71.62, 60.38, 46.29, 30.85, 28.31, 23.45, 19.76; *m*/*z* (ESI; 99%) calcd for C_27_H_32_N_2_O_9_ = 596.22 found 597.2 [M + H]^+^.

##### 1-((l-Prolyl)oxy)ethyl-4-(2-hydroxy-2,2-diphenylacetoxy)piperidine-1-carboxylate Hydrochloride **9**

The title compound was synthesized according to General Chemistry Procedure **3** from **9i** to obtain the title compound **9** (quantitative) as a white solid. ^1^H (400 MHz; D_6_-DMSO) δ 10.2 (1H, bs), 9.05 (1H, bs), 7.40–7.29 (10H, m), 6.72 (1H, q, *J* = 5 Hz), 5.06 (1H, s), 4.39 (1H, bs), 4.29 (1H, bs), 3.97 (1H, s), 3.44–3.30 (4H, m), 2.33–2.16 (1H, m), 2.08–1.69 (6H, m), 1.58–1.47 (5H, m); ^13^C (101 MHz, D_6_-DMSO) δ 172.75, 170.87, 152.58, 143.77, 128.31, 128.27, 127.95, 127.51, 91.37, 81.18, 70.32, 59.10, 45.84, 45.75, 28.39, 28.32, 28.28, 23.58, 23.35, 19.82; *m*/*z* (ESI; 99%) calcd for C_27_H_32_N_2_O_7_ = 496.56 found 497.3 [M + H]^+^.

##### 1-(((*tert*-Butoxycarbonyl)-l-alanyl)oxy)ethyl-4-(2-hydroxy-2,2-diphenylacetoxy)piperidine-1-carboxylate **10i**

The title compound was synthesized according to General Chemistry Procedure **1** using (*tert*-butoxycarbonyl)-l-alanine and purified using flash column chromatography using a gradient of 20–80% EtOAc:(40–60) PET to obtain compound **10i** (49%) as a colorless oil. ^1^H (400 MHz; CDCl_3_) δ 7.48–7.29 (10H, m), 6.77 (1H, q, *J* = 5 Hz), 5.21–5.04 (2H, m), 4.30 (2H, m), 3.66 (1H, bs), 3.51–3.16 (4H, m), 1.91–1.77 (2H, m), 1.72–1.61 (2H, m), 1.51–1.47 (3H, m), 1.36 (3H, dd, *J* = 7 and 11 Hz) 1.44 (9H, s); ^13^C (101 MHz; CDCl_3_) δ 173.84, 171.71, 155.05, 152.72, 141.85, 128.15, 127.38, 90.68, 81.06, 79.92, 71.65, 49.14, 40.91, 40.44, 29.80, 29.78, 28.32, 18.37, 17.29; *m*/*z* (ESI; 99%) calcd for C_30_H_38_N_2_O_9_ = 570.64 found 571.3 [M + H]^+^.

##### 1-((l-Alanyl)oxy)ethyl-4-(2-hydroxy-2,2-diphenylacetoxy)piperidine-1-carboxylate Hydrochloride **10**

The title compound was synthesized according to General Chemistry Procedure **3** from **10i** to obtain the title compound **10** (quantitative) as a white solid. ^1^H (400 MHz; D_6_-DMSO) δ 8.25 (3H, bs), 7.48–7.29 (10H, m), 6.71 (1H, q, *J* = 5 Hz), 5.08–5.03 (1H, m), 4.14–3.69 (4H, m), 3.45–3.14 (2H, m), 1.84–1.70 (2H, m), 1.60–1.44 (5H, m), 1.35 (3H, dd, *J* = 7 and 11 Hz); ^13^C (101 MHz; D_6_-DMSO) δ 172.75, 168.86, 152.50, 143.77, 129.14, 128.31, 128.27, 127.99, 127.95, 127.62, 127.51, 127.41, 91.14, 81.18, 70.32, 48.28, 48.17, 19.85, 16.22; *m*/*z* (ESI; 99%) calcd for C_25_H_30_N_2_O_7_ = 470.52 found 471.2 [M + H]^+^.

##### 1-((2-((*tert*-Butoxycarbonyl)amino)-2-methylpropanoyl)oxy)ethyl-4-(2-hydroxy-2,2-diphenylacetoxy)piperidine-1-carboxylate **11i**

The title compound was synthesized according to General Chemistry Procedure **1** using 2-((*tert*-butoxycarbonyl)amino)-2-methylpropanoic acid and purified using flash column chromatography using a gradient of 20–80% EtOAc:(40–60) PET to obtain the title compound **11i** (49%) as a colorless oil. ^1^H (400 MHz; CDCl_3_) δ 7.46–7.29 (10H, m), 6.68 (1H, q, *J* = 5 Hz), 5.16–5.12 (1H, m), 5.04 (1H, bs), 3.66 (1H, bs), 3.51–3.20 (4H, m), 1.92–1.77 (2H, m), 1.72–1.61 (2H, m), 1.50–1.45 (9H, m), 1.43 (9H, s); ^13^C (101 MHz, CDCl_3_) δ 173.86, 172.84, 154.47, 152.87, 141.85, 128.15, 127.38, 90.88, 81.05, 79.86, 71.81, 55.96, 40.91, 40.49, 33.84, 30.05, 28.32, 25.03, 19.72; *m*/*z* (ESI; 99%) calcd for C_31_H_40_N_2_O_9_ = 584.67 found 585.3 [M + H]^+^.

##### 1-((2-Amino-2-methylpropanoyl)oxy)ethyl-4-(2-hydroxy-2,2-diphenylacetoxy)piperidine-1-carboxylate Hydrochloride **11**

The title compound was synthesized according to General Chemistry Procedure **3** from **11i** to obtain the title compound **11** (quantitative) as a white solid. ^1^H (400 MHz; D_6_-DMSO) δ 8.73 (3H, bs), 7.46–7.29 (10H, m), 6.69 (1H, q, *J* = 5 Hz), 5.08–5.03 (1H, m), 5.05 (1H, bs), 3.53–3.18 (4H, m), 1.57–1.39 (9H, m), 1.92–1.77 (2H, m), 1.85–1.72 (2H, m); ^13^C (101 MHz, D_6_-DMSO) δ 173.83, 172.74, 152.50, 143.77, 129.14, 128.26, 127.94, 127.61, 127.51, 91.38, 81.17, 70.36, 56.21, 30.19, 29.90, 19.73; *m*/*z* (ESI; 99%) calcd for C_26_H_32_N_2_O_7_ = 484.55 found 485.6 [M + H]^+^.

##### 1-(((*S*)-2-((*tert*-Butoxycarbonyl)amino)butanoyl)oxy)ethyl-4-(2-hydroxy-2,2-diphenylacetoxy)piperidine-1-carboxylate **12i**

The title compound was synthesized according to General Chemistry Procedure **1** using (*S*)-2-((*tert*-butoxycarbonyl)amino)butanoic acid and purified using flash column chromatography using a gradient of 20–80% EtOAc:(40–60) PET to obtain the title compound **12i** (54%) as a colorless oil. ^1^H (400 MHz; CDCl_3_) δ 7.47–7.30 (10H, m), 6.82 (1H, q, *J* = 5 Hz), 5.16 (1H, bs), 5.07 (1H, bs), 4.32–4.20 (2H, m), 3.52–3.32 (4H, m), 1.90–1.77 (2H, m), 1.74–1.60 (4H, m), 1.52–1.47 (3H, m), 1.45 (9H, s), 0.96–0.90 (2H, m); ^13^C (101 MHz; CDCl_3_) δ 173.85, 171.11, 155.39, 152.67, 141.84, 128.16, 127.38, 90.59, 81.05, 77.40, 54.39, 43.63, 30.04, 28.32, 23.87, 19.77, 9.38; *m*/*z* (ESI; 97%) calcd for C_31_H_40_N_2_O_9_ = 584.67 found 585.3 [M + H]^+^.

##### 1-(((*S*)-2-Aminobutanoyl)oxy)ethyl-4-(2-hydroxy-2,2-diphenylacetoxy)piperidine-1-carboxylate Hydrochloride **12**

The title compound was synthesized according to General Chemistry Procedure **3** from **12i** to obtain the title compound **12** (quantitative) as a white solid. ^1^H (400 MHz; D_6_-DMSO) δ 8.61 (3H, bs), 7.47–7.30 (10H, m), 6.76 (1H, q, *J* = 5 Hz), 5.08–5.03 (1H, m), 4.00 (1H, bs), 3.44–3.20 (4H, m), 1.89–1.69 (4H, m), 1.57–1.44 (5H, m), 0.96–0.82 (3H, m); ^13^C (101 MHz; D_6_-DMSO) δ 172.73, 171.35, 152.44, 143.77, 128.26, 127.94, 127.51, 127.41, 91.13, 81.17, 65.39, 53.43, 53.19, 23.74, 23.70, 19.88, 19.86, 9.10; *m*/*z* (ESI; 99%) calcd for C_26_H_32_N_2_O_7_ = 484.55 found 485.3 [M + H]^+^.

##### 1-(((*tert*-Butoxycarbonyl)-l-leucyl)oxy)ethyl-4-(2-hydroxy-2,2-diphenylacetoxy)piperidine-1-carboxylate **13i**

The title compound was synthesized according to General Chemistry Procedure **1** using (*tert*-butoxycarbonyl)-l-leucine and purified using flash column chromatography using a gradient of 20–80% EtOAc:(40–60) PET to obtain the title compound **13i** (46%) as a colorless oil. ^1^H (400 MHz; CDCl_3_) δ 7.47–7.30 (10H, m), 6.82 (1H, q, *J* = 5 Hz), 5.17 (1H, bs), 4.93 (1H, bs), 4.35–4.23 (2H, m), 3.52–3.16 (4H, m), 1.89–1.78 (2H, m), 1.75–1.61 (4H, m), 1.60–1.56 (1H, m), 1.52–1.47(3H, m), 1.44 (9H, s), 0.98–0.91 (6H, m); ^13^C (101 MHz; D_6_-DMSO) δ 171.48, 168.72, 144.67, 130.19, 129.40, 129.33, 129.05, 128.50, 128.42, 82.22, 62.40, 41.38, 41.17, 40.96, 40.75, 40.55, 40.34, 40.13, 34.66, 34.15, 27.75, 27.46, 20.82; *m*/*z* (ESI; 99%) calcd for C_33_H_44_N_2_O_9_ = 612.72 found 613.3 [M + H]^+^.

##### 1-((l-Leucyl)oxy)ethyl-4-(2-hydroxy-2,2-diphenylacetoxy)piperidine-1-carboxylate Hydrochloride **13**

The title compound was synthesized according to General Chemistry Procedure **3** from **13i** to obtain the title compound **13** (quantitative) as a white solid. ^1^H (400 MHz; D_6_-DMSO) δ 8.59 (3H, bs) 7.47–7.30 (10H, m), 6.74 (1H, q, *J* = 5 Hz), 5.08–5.03 (1H, m), 3.99–3.86 (1H, m), 3.43–3.15 (5H, m), 1.85–1.37 (10H, m), 1.08–0.97 (6H, m); ^13^C (101 MHz; D_6_-DMSO) δ 172.75, 168.98, 158.90, 143.76, 129.13, 128.30, 128.26, 127.94, 127.52, 127.41, 117.53, 114.64, 91.15, 81.17, 70.36, 50.91, 50.81, 29.88, 24.20, 24.15, 22.56, 19.80; *m*/*z* (ESI; 99%) calcd for C_28_H_36_N_2_O_7_ = 512.60 found 513.4 [M + H]^+^.

##### 1-(((*tert*-Butoxycarbonyl)-l-valyl)oxy)ethyl-4-(2-hydroxy-2,2-diphenylacetoxy)piperidine-1-carboxylate **14i**

The title compound was synthesized according to General Chemistry Procedure **1** using (*tert*-butoxycarbonyl)-l-valine and purified using flash column chromatography using a gradient of 20–80% EtOAc:(40–60) PET to obtain the title compound **14i** (41%) as a colorless oil. ^1^H (400 MHz; CDCl_3_) δ 7.47–7.26 (10H, m), 6.62 (1H, q, *J* = 5 Hz), 5.15 (1H, bs), 5.07 (1H, bs), 4.20 (1H, m), 3.49–3.12 (4H, m), 2.11 (1H, m), 1.87–1.72 (2H, m), 1.70–1.57 (2H, m), 1.50–1.40 (12H, m), 1.45 (9H, s), 0.98–0.82 (6H, m); ^13^C (101 MHz; CDCl_3_) δ 173.76, 171.15, 157.58, 155.68, 141.89, 128.10, 127.36, 120.21, 90.49, 82.35, 81.04, 71.59, 60.38, 29.78, 28.48, 28.30, 20.88; *m*/*z* (ESI; 96%) calcd for C_32_H_42_N_2_O_9_ = 598.69 found 599.0 [M + H]^+^.

##### 1-((l-Valyl)oxy)ethyl-4-(2-hydroxy-2,2-diphenylacetoxy)piperidine-1-carboxylate Hydrochloride **14**

The title compound was synthesized according to General Chemistry Procedure **3** from **14i** to obtain the title compound **14** (quantitative) as a white solid. ^1^H (400 MHz); D_6_-DMSO δ 8.22 (3H, bs), 7.47–7.31 (10H, m), 6.67 (1H, q, *J* = 5 Hz), 5.14 (1H, bs), 3.92–3.71 (2H, m), 3.48–3.16 (4H, m), 3.09–2.89 (1H, m), 1.92–1.22 (2H, m), 1.85–1.66 (2H, m), 1.59–1.44 (3H, m), 1.00–0.84 (9H, m); ^13^C (101 MHz; D_6_-DMSO) δ 170.76, 170.43, 166.22, 143.65, 126.35, 128.29, 128.06, 126.00, 127.47, 127.38, 81.19, 73.60, 65.39, 30.80, 29.50, 19.80, 19.46; *m*/*z* (ESI; 99%) calcd for C_27_H_34_N_2_O_7_ = 498.58 found 499.3 [M + H]^+^.

##### 1-(((*S*)-2-((*tert*-Butoxycarbonyl)amino)-3,3-dimethylbutanoyl)oxy)ethyl-4-(2-hydroxy-2,2-diphenylacetoxy)piperidine-1-carboxylate **15i**

The title compound was synthesized according to General Chemistry Procedure **1** and purified using flash column chromatography using a gradient of 20–80% EtOAc:(40–60) PET to obtain the title compound **15i** (75%) as a colorless oil. ^1^H (400 MHz; CDCl_3_) δ 7.47–7.30 (10H, m), 6.83 (1H, q, *J* = 5 Hz), 5.14 (1H, bs), 4.22 (1H, d, *J* = 3 Hz), 4.15–4.02 (1H, bs), 3.51–3.23 (2H, m), 3.22–3.07 (1H, m), 1.88–1.72 (2H, m), 1.70–1.52 (2H, m), 1.51–1.40 (14H, m), 0.96 (9H, s); ^13^C (101 MHz; CDCl_3_) 173.77, 171.17, 157.58, 155.67, 141.87, 137.98, 128.10, 127.36, 121.11, 90.39, 84.98, 82.42, 62.28, 40.97, 38.60,, 29.67, 29.33, 28.47, 28.38, 19.88; *m*/*z* (ESI; 98%) calcd for C_33_H_44_N_2_O_9_ = 612.72 found 613.3 [M + H]^+^.

##### 1-(((*S*)-2-Amino-3,3-dimethylbutanoyl)oxy)ethyl-4-(2-hydroxy-2,2-diphenylacetoxy)piperidine-1-carboxylate Hydrochloride **15**

The title compound was synthesized according to General Chemistry Procedure **3** from **15i** to obtain the title compound **15** (quantitative) as a white solid. ^1^H (400 MHz; D_6_-DMSO) δ 8.19 (3H, bs), 7.47–7.29 (10H, m), 6.82 (1H, q, *J* = 5 Hz), 5.08–5.03 (1H, m), 3.42–3.10 (4H, m), 3.09–2.84 (1H, m), 1.85–1.74 (2H, m), 1.85–1.61 (2H, m), 1.57–1.42 (3H, m), 0.97 (9H, 2s); ^13^C (101 MHz; D_6_-DMSO) δ 173.76, 170.58, 155.54, 152.81, 141.82, 128.11, 127.31, 90.36, 81.02, 79.69, 71.76, 66.38, 44.52, 43.46, 30.01, 28.35, 26.40, 17.02; *m*/*z* (ESI; 99%) calcd for C_28_H_36_N_2_O_7_ = 512.60 found 513.3 [M + H]^+^.

##### 1-(((*tert*-Butoxycarbonyl)-l-isoleucyl)oxy)ethyl-4-(2-hydroxy-2,2-diphenylacetoxy)piperidine-1-carboxylate **16i**

The title compound was synthesized according to General Chemistry Procedure **1** using (*tert*-butoxycarbonyl)-l-isoleucine and purified using flash column chromatography using a gradient of 20–80% EtOAc:(40–60) PET to obtain the title compound **16i** (35%) as a colorless oil. ^1^H (400 MHz; CDCl_3_) δ 7.46–7.31 (10H, m), 6.84 (1H, q, *J* = 5 Hz), 5.17 (1H, bs), 5.07 (1H, bs), 4.37–4.21 (2H, m), 3.70–3.61 (1H, bs), 3.52–3.13 (4H, m), 1.78–1.58 (2H, m), 1.57–1.52 (2H, m), 1.51–1.47 (3H, m), 1.43 (9H, s), 0.95–0.85 (8H, s); ^13^C (101 MHz; CDCl_3_) δ 173.83, 170.66, 155.61, 152.57, 141.86, 128.14, 127.38, 90.36, 81.05, 79.83, 71.72, 57.75, 40.93, 40.43, 37.92, 29.69, 28.32, 24.97, 19.86, 15.43, 11.70; *m*/*z* (ESI; 97%) calcd for C_33_H_44_N_2_O_9_ = 612.72 found 613.7 [M + H]^+^.

##### 1-((l-Isoleucyl)oxy)ethyl-4-(2-hydroxy-2,2-diphenylacetoxy)piperidine-1-carboxylate Hydrochloride **16**

The title compound was synthesized according to General Chemistry Procedure **3** from 1**6i** to obtain the title compound **16** (quantitative) as a white solid. ^1^H (400 MHz; D_6_-DMSO) δ 8.57 (2H, bs), 7.46–7.31 (10H, m), 6.74 (1H, q, *J* = 5 Hz), 5.17 (1H, bs), 5.08–5.03 (1H, bs), 3.94 (1H, bs), 3.42–3.21 (4H, m), 1.95–1.85 (2H, m), 1.84–1.72 (2H, m), 1.60–1.34 (7H, m), 0.96–0.75 (6H, s); ^13^C (101 MHz; D_6_-DMSO) δ 172.75, 167.18, 159.05, 143.77, 129.13, 128.26, 127.94, 127.52, 127.41, 91.10, 81.17, 70.36, 56.35, 36.43, 25.69, 19.94, 19.82, 14.55, 12.02, 11.98; *m*/*z* (ESI; 98%) calcd for C_28_H_36_N_2_O_7_ = 512.60 found 513.2 [M + H]^+^.

##### 1-((l-Lysyl)oxy)ethyl-4-(2-hydroxy-2,2-diphenylacetoxy)piperidine-1-carboxylate **17i**

The title compound was synthesized according to General Chemistry Procedure **1** using *N*,*N*-bis(*tert*-butoxycarbonyl)-l-lysine to obtain the title compound **17i** (75%) as a colorless oil. ^1^H (400 MHz; CDCl_3_) δ 1.31–1.54 (24H, m), 1.58–1.72 (2H, m), 1.77–1.88 (2H, m), 3.10–3.47 (5H, m), 4.28 (1H, bs), 5.20–5.04 (4H, m), 5.07 (1H, bs), 6.82 (1H, q, *J* = 5 Hz), 7.31–7.46 (10H, m); ^13^C (101 MHz, D_6_-DMSO) δ 172.75, 152.29, 143.77, 143.74, 129.13, 128.26, 127.94, 127.52, 127.41, 91.10, 81.17, 70.36, 56.35, 40.61, 40.45, 40.40, 40.25, 40.20, 39.99, 39.78, 39.57, 39.36, 36.43, 25.69, 19.94, 19.82, 14.55, 12.02, 11.98; *m*/*z* (ESI; 99%) calcd for C_38_H_53_N_3_O_11_ = 727.85 found 728.4 [M + H]^+^.

##### 1-((l-Lysyl)oxy)ethyl-4-(2-hydroxy-2,2-diphenylacetoxy)piperidine-1-carboxylate Dihydrochloride **17**

The title compound was synthesized according to General Chemistry Procedure **3** from **17i** to obtain the title compound **17** (quantitative) as a white solid.^1^H (400 MHz; D_6_-DMSO) δ 8.65 (3H, bs), 8.03 (3H, bs), 7.46–7.31 (10H, m), 6.74 (1H, q, *J* = 5 Hz), 5.08–5.03 (1H, m), 4.06–3.96 (1H, m), 3.42–3.15 (2H, m), 2.75–2.65 (4H, m), 1.85–1.71 (4H, m), 1.82–1.28 (9H, m); ^13^C (101 MHz, D_6_-DMSO) δ 172.76, 168.49, 158.91, 143.77, 129.15, 128.27, 127.95, 127.51, 91.22, 81.18, 70.31, 51.94, 38.63, 29.65, 29.57, 26.69, 26.63, 21.45, 19.84; *m*/*z* (ESI; 99%) calcd for C_28_H_37_N_3_O_7_ = 527.62 found 528.2 [M + H]^+^.

##### 1-(((*R*)-2-((*tert*-Butoxycarbonyl)amino)-3,3-dimethylbutanoyl)oxy)ethyl-4-(2-hydroxy-2,2-diphenylacetoxy)piperidine-1-carboxylate **18i**

The title compound was synthesized according to General Chemistry Procedure **1** using (*R*)-2-((*tert*-butoxycarbonyl)amino)-3,3-dimethylbutanoic acid and purified using flash column chromatography using a gradient of 20–80% EtOAc:(40–60) PET to obtain the title compound **18i** (75%) as a colorless oil. ^1^H (400 MHz; D_6_-DMSO) δ 7.47–7.29 (10H, m), 6.85 (1H, q, *J* = 5 Hz), 5.11 (1H, bs), 4.22 (1H, m), 4.05 (1H, bs), 3.68–3.58 (2H, m), 3.52–3.28 (2H, m), 3.17 (1H, m), 1.85–1.74 (2H, m), 1.69–1.56 (2H, m), 1.52–1.45 (3H, m), 1.43 (9H, s), 0.97 (9H, s); ^13^C (101 MHz; D_6_-DMSO) δ 171.48, 168.72, 152.25, 144.67, 130.19, 129.40, 129.33, 129.05, 128.50, 128.42, 94.88, 82.22, 62.40, 41.38, 27.75, 27.46, 20.82; *m*/*z* (ESI; 98%) calcd for C_33_H_44_N_2_O_9_ = 612.72 found 613.3 [M + H]^+^.

##### 1-(((*S*)-2-Amino-3,3-dimethylbutanoyl)oxy)ethyl-4-(2-hydroxy-2,2-diphenylacetoxy)piperidine-1-carboxylate Hydrochloride **18**

The title compound was synthesized according to General Chemistry Procedure **3** from **18i** to obtain the title compound **18** (quantitative) as a white solid. ^1^H (400 MHz; D_6_-DMSO) δ 8.19 (3H, bs), 7.47–7.30 (10H, m), 6.77 (1H, q, *J* = 5 Hz), 5.04 (1H, bs), 3.92–3.71 (2H, m), 3.44–3.10 (4H, m), 3.02–2.86 (1H, m), 1.85–1.74 (2H, m), 1.85–1.61 (2H, m), 1.57–1.42 (3H, m), 1.04–0.91 (9H, s); ^13^C (101 MHz; D_6_-DMSO) δ 173.76, 170.42, 155.55, 152.81, 141.89, 128.11, 127.36, 90.37, 81.03, 79.77, 71.72, 66.37, 44.42, 43.86, 30.03, 28.29, 26.42, 17.50; *m*/*z* (ESI; 99%) calcd for C_28_H_36_N_2_O_7_ = 512.60 found 513.3 [M + H]^+^.

##### 1-(((*tert*-Butoxycarbonyl)-d-valyl)oxy)ethyl-4-(2-hydroxy-2,2-diphenylacetoxy)piperidine-1-carboxylate **19i**

The title compound was synthesized according to General Chemistry Procedure **1** using (*tert*-butoxycarbonyl)-d-valine and purified using flash column chromatography using a gradient of 20–80% EtOAc:(40–60) PET to obtain the title compound **19i** (41%) as a colorless oil. ^1^H (400 MHz; CDCl_3_) δ 7.47–7.30 (10H, m), 6.85 (1H, q, *J* = 5 Hz), 5.17 (1H, bs), 5.06 (1H, bs), 4.22 (2H, d, *J* = 3 Hz), 3.52–3.17 (4H, m), 2.13 (1H, m), 1.91–1.77 (2H, m), 1.71–1.59 (2H, m), 1.52–1.47 (3H, m), 1.45 (9H, s), 1.00–0.50 (6H, ddd, *J* = 3, 7 and 30 Hz); ^13^C (101 MHz; CDCl_3_) δ 173.85, 170.75, 155.71, 152.63, 141.85, 128.15, 127.38, 90.53, 81.06, 79.87, 71.67, 58.26, 40.91, 40.44, 30.07, 29.75, 28.31, 18.87, 17.30; *m*/*z* (ESI; 96%) calcd for C_32_H_42_N_2_O_9_ = 598.69 found 599.0 [M + H]^+^.

##### 1-((d-Valyl)oxy)ethyl-4-(2-hydroxy-2,2-diphenylacetoxy)piperidine-1-carboxylate Hydrochloride **19**

The title compound was synthesized according to General Chemistry Procedure **3** from **19i** to obtain the title compound **19** (quantitative) as a white solid. ^1^H (400 MHz; D_6_-DMSO) δ 8.24 (3H, bs), 7.47–7.30 (10H, m), 6.77 (1H, q, *J* = 5 Hz), 5.20–5.04 (1H, m), 3.92–3.72 (2H, m), 3.48–3.16 (4H, m), 3.09–2.89 (1H, m), 1.22–1.93 (2H, m), 1.85–1.66 (2H, m), 1.59–1.43 (3H, m), 1.00–0.86 (6H, m); ^13^C (101 MHz; D_6_-DMSO) δ 170.76, 169.43, 166.22, 143.65, 128.35, 128.29, 128.06, 128.00, 127.47, 127.38, 81.19, 74.60, 65.39, 29.80, 29.50, 19.80, 18.46; *m*/*z* (ESI; 99%) calcd for C_27_H_34_N_2_O_7_ = 498.58 found 499.3 [M + H]^+^.

##### 1-(((*S*)-3,7-Bis((*tert*-butoxycarbonyl)amino)heptanoyl)oxy)ethyl-4-(2-hydroxy-2,2-diphenylacetoxy)piperidine-1-carboxylate **20i**

(a) 1-(((*S*)-7-(((Benzyloxy)carbonyl)amino)-3-((*tert*-butoxycarbonyl)amino) heptanoyl)oxy) ethyl 4-(2-hydroxy-2,2-diphenylacetoxy) piperidine-1-carboxylate was synthesized according to General Chemistry Procedure **1** using (*S*)-7-(((benzyloxy)carbonyl)amino)-3-((*tert*-butoxycarbonyl)amino)heptanoic acid to obtain 1-(((*S*)-7-(((benzyloxy)carbonyl)amino)-3-((*tert*-butoxycarbonyl)amino) heptanoyl)oxy) ethyl 4-(2-hydroxy-2,2-diphenylacetoxy) piperidine-1-carboxylate as a pale yellow oil (72%). ^1^H (400 MHz; CDCl_3_) δ 7.29–7.26 [15H, m], 6.75 (1H, q, *J* = 5 Hz), 5.11–5.05 (1H, m); 5.04 (2H, s), 3.89 (1H, bs), 3.44–3.35 (2H, m), 3.34–3.24 (2H, m), 3.21–3.12 (2H, m), 2.47–2.53 (2H, m), 1.76–1.86 (2H, m), 1.60–1.70 (2H, m), 1.60–1.70 (2H, m), 1.49–1.58 (4H, m), 1.47 (3H, d, *J* = 5 Hz), 1.42 (9H, s), 1.37 (2H, m); ^13^C (101 MHz; CDCl_3_) δ 174.87, 173.81, 156.62, 155.60, 152.92, 141.85, 136.61, 128.49, 128.14, 128.07, 127.36, 90.19, 81.08, 79.43, 47.27, 47.22, 42.21, 40.91, 31.57, 30.04, 29.77, 28.37, 21.04, 19.68; *m*/*z* (ESI; 99%) calcd for C_42_H_53_N_3_O_11_ = 775.90 found 776.5 [M + H].

(b) The product from step (a) (0.050 g, 0.064 mmol) was dissolved in MeOH (15 mL), and 10% Pd/C (10 mg) was added. The flask was placed under an atmosphere of hydrogen for 2.5 h before the palladium was filtered through a celite pad. The filtrate was concentrated, dissolved in DCM (15 mL), and di-*tert*-butyl dicarbonate (50 mg) and NEt_3_ (27 μL, 3 equiv) were added, and the solution was left to stir for 2 h. The solution was then concentrated, and the residue was purified using a gradient of 40% EtOAc in Pet to achieve the title compound **20i** (75%) as a colorless oil. ^1^H (400 MHz; CDCl_3_) δ 7.46–7.31 (10H, m), 6.77 (1H, q, *J* = 5 Hz), 5.17 (1H, bs), 5.18 (1H, bs), 4.25 (1H, bs), 3.95–3.82 (1H, m), 3.47–3.37 (2H, m), 3.36–3.25 (2H, m), 3.15–3.04 (2H, m), 2.56–2.48 (2H, m), 1.90–1.79 (2H, m), 1.72–1.62 (3H, m), 1.57–1.30 (30H, m); ^13^C (101 MHz; CDCl_3_) δ 173.84, 156.06, 155.82, 141.82, 128.16, 127.36, 90.09, 81.03, 78.98, 48.45, 47.35, 40.43, 40.16, 28.45, 28.42, 28.40, 23.22, 19.82; *m*/*z* (ESI; 99%) calcd for C_39_H_55_N_3_O_11_ = 741.88 found 742.4 [M + H]^+^.

##### 1-(((*S*)-3,7-Diaminoheptanoyl)oxy)ethyl-4-(2-hydroxy-2,2-diphenylacetoxy)piperidine-1-carboxylate Dihydrochloride **20**

The title compound was synthesized according to General Chemistry Procedure **3** from **20i** to obtain the title compound **20** as a mixture of diastereoisomers as a white solid. ^1^H (400 MHz; D_6_-DMSO) δ 8.21 (3H, bs), 7.98 (3H, bs), 7.46–7.31 (10H, m), 6.67 (1H, q, *J* = 5 Hz), 5.05 (1H, bs), 3.36–3.25 (2H, m), 3.44–3.17 (4H, m), 2.80–2.70 (1H, m), 1.82–1.70 (2H, m), 1.68–1.47 (7H, m), 1.46–1.34 (5H, m); ^13^C (101 MHz; D_6_-DMSO) δ 173.84, 156.06, 155.82, 141.82, 128.16, 127.36, 90.09, 81.03, 78.98, 48.45, 47.35, 40.43, 40.16, 28.45, 28.42, 28.40, 23.22, 19.82; *m*/*z* (ESI; 99%) calcd for C_29_H_39_N_3_O_7_ = 541.65 found 542.4 [M + H]^+^.

##### (10*R*)-10-((*tert*-Butoxycarbonyl)amino)-2,2-dimethyl-4,11,15-trioxo-3,16-dioxa-5,12-diazaoctadecan-17-yl-4-(2-hydroxy-2,2-diphenylacetoxy)piperidine-1-carboxylate **21i**

The title compound was synthesized according to General Chemistry Procedure **2** from **4** and *N*,*N*-bis(*tert*-butoxycarbonyl)-l-lysine and purified using flash column chromatography using a gradient of 20–80% EtOAc:(40–60) PET to obtain the title compound **21i** as a mixture of diastereoisomers (37%) as a colorless oil. ^1^H (400 MHz; CDCl_3_) δ 7.47–7.30 (10H, m), 6.74 (1H, q, *J* = 5 Hz), 5.22–5.09 (2H, m), 4.55 (1H, m), 4.05 (1H, bs), 3.51–3.06 (6H, m), 2.58–2.48 (2H, m), 1.92–1.75 (3H, m), 1.74–1.63 (3H, m), 1.62–1.532 (2H, m), 1.49 (3H,d, *J* = 5 Hz), 1.44 (18H, s), 1.40–1.29 (2H, m); ^13^C (101 MHz; CDCl_3_) δ 173.83, 171.18, 169.89, 156.18, 152.56, 141.81, 128.14, 127.35, 127.10, 90.46, 82.78, 81.04, 71.69, 60.41, 40.88, 40.45, 29.69, 28.44, 28.30, 23.86, 22.62, 14.20, 11.58; *m*/*z* (ESI; 99%) calcd for C_41_H_58_N_4_O_12_ = 798.93 found 799.4 [M + H]^+^.

##### 1-((3-((*R*)-2,6-Diaminohexanamido)propanoyl)oxy)ethyl-4-(2-hydroxy-2,2-diphenylacetoxy)piperidine-1-carboxylate Dihydrochloride **21**

The title compound was synthesized according to General Chemistry Procedure **3** from **21i** to obtain the title compound **21**(quantitative) as a white solid. ^1^H (400 MHz; D_6_-DMSO) δ 8.60 (1H, t, J=7.2 Hz), 7.90 (6H, bs), 7.47–7.30 (10H, m), 6.62 (1H, q, *J* = 5 Hz), 5.08–5.04 (1H, m), 4.23–4.12 (1H, m), 3.40–3.16 (3H, m), 3.01–2.90 (2H, m), 2.80–2.66 (2H, m), 2.59–2.52 (2H, m), 1.83–1.70 (2H, m), 1.69–1.45 (6H, m), 1.45–1.30 (6H, m); ^13^C (101 MHz, D_6_-DMSO) δ 173.83, 171.18, 156.68, 140.81, 128.24, 126.35, 127.10, 90.56, 82.75, 71.54, 60.42, 40.72, 40.10, 29.41, 28.25, 28.34, 23.86, 22.62, 14.20, 11.58. *m*/*z* (ESI; 99%) calcd for C_31_H_42_N_4_O_8_ = 598.70 found 599.2 [M + H]^+^.

##### (10*S*,13*S*)-10-((*tert*-Butoxycarbonyl)amino)-13-(*tert*-butyl)-2,2-dimethyl-4,11,14-trioxo-3,15-dioxa-5,12-diazaheptadecan-16-yl-4-(2-hydroxy-2,2-diphenylacetoxy)piperidine-1-carboxylate **22i**

The title compound was synthesized according to General Chemistry Procedure **2** from **15** and *N*,*N*-bis(*tert*-butoxycarbonyl)-l-lysine and was purified using flash column chromatography using a gradient of 20–80% EtOAc:(40–60) PET to obtain the title compound **22i** as a mixture of diastereoisomers (94%) as a colorless oil. ^1^H (400 MHz; CDCl_3_) δ 7.47–7.30 (10H, m), 6.81 (1H, q, *J* = 5 Hz), 5.32 (4H, m), 5.18(1H, bs), 4.83–4.64 (1H, m), 4.06 (1H, bs), 3.66 (1H, bs), 3.72–3.29 (4H, m), 3.15–3.05 (2H, m), 1.88–1.75 (3H, m), 1.70–1.59 (3H, m), 1.58–1.50 (2H, m), 1.50–1.46 (3H,m), 1.45 (18H, s), 1.37–1.30 (2H, m), 1.02–0.94 (9H, m); 13C (101 MHz, CDCl_3_) δ 173.74, 172.33, 171.15, 156.18, 155.81, 152.55, 141.91, 128.08, 127.34, 90.51, 82.41, 81.05, 71.54, 60.36, 40.94, 40.42, 39.86, 33.81, 30.95, 30.04, 29.58, 28.40, 27.62, 23.90, 22.57, 18.68; *m*/*z* (ESI; 99%) calcd for C_44_H_64_N_4_O_12_ = 841.01 found 841.4 [M + H]^+^.

##### 1-(((*S*)-2-((*S*)-2,6-Diaminohexanamido)-3,3-dimethylbutanoyl)oxy)ethyl-4-(2-hydroxy-2,2-diphenylacetoxy)piperidine-1-carboxylate Dihydrochloride **22**

The title compound was synthesized according to General Chemistry Procedure **3** from **22i** to obtain the title compound **22** (quantitative) as a white solid. ^1^H (400 MHz; D_6_-DMSO) δ 8.34 (2H, bs), 8.06 (2H, bs), 7.47–7.30 (10H, m), 6.69 (1H, q, *J* = 5 Hz), 5.04 (1H, bs), 4.12–4.00 (2H, m), 3.42–3.29 (4H, m), 3.15–3.05 (2H, m), 3.41– 3.15 (2H, m), 2.79–2.70 (2H, m), 1.77–1.67 (2H, m), 1.63–1.43 (4H, m), 1.44–1.34 (6H, m), 0.97 (9H, s); ^13^C (101 MHz, D_6_-DMSO) δ 172.74, 172.72, 169.67, 159.38, 143.76, 129.11, 128.24, 127.92, 127.51, 120.28 90.20, 81.17, 70.39, 65.37, 51.83, 38.69, 33.93, 33.91, 30.98, 30.90, 30.26, 28.82, 19.92; *m*/*z* (ESI; 99%) calcd for C_34_H_48_N_4_O_8_ = 640.78 found 642.2 [M + H]^+^.

##### (10*S*,13*R*)-10-((*tert*-Butoxycarbonyl)amino)-13-(*tert*-butyl)-2,2-dimethyl-4,11,14-trioxo-3,15-dioxa-5,12-diazaheptadecan-16-yl-4-(2-hydroxy-2,2-diphenylacetoxy)piperidine-1-carboxylate **23i**

The title compound was synthesized according to General Chemistry Procedure **2** from **18** and *N*,*N*-bis(*tert*-butoxycarbonyl)-l-lysine and purified using flash column chromatography using a gradient of 20–80% EtOAc:(40–60) PET to obtain the title compound **23i** as a mixture of diastereoisomers (86%) as a colorless oil. ^1^H (400 MHz; CDCl_3_) δ 7.47–7.30 (10H, m), 6.81 (1H, q, *J* = 5 Hz), 5.32 (4H, m), 5.18(1H, bs), 4.83–4.64 (1H,m), 4.06 (1H, bs), 3.66 (1H, bs), 3.72–3.29 (6H, m), 3.15–3.05 (2H, m), 1.88–1.75 (3H, m), 1.70–1.59 (3H, m), 1.58–1.50 (2H, m), 1.50–1.46 (3H, m), 1.45 (18H, s) 1.37–1.30 (2H, m), 1.02–0.94 (9H, m); ^13^C (101 MHz, CDCl_3_) δ 173.74, 172.33, 171.15, 156.18, 155.81, 152.55, 141.91, 128.08, 127.34, 90.51, 82.41, 81.05, 71.54, 60.36, 40.94, 40.42, 39.86, 33.81, 30.95, 30.04, 29.58, 28.40, 27.62, 23.90, 22.57, 18.68; *m*/*z* (ESI; 99%) calcd for C_44_H_61_N_4_O_12_ = 841.01 found 841.5 [M + H]^+^.

##### 1-(((*R*)-2-((*S*)-2,6-Diaminohexanamido)-3,3-dimethylbutanoyl)oxy)ethyl-4-(2-hydroxy-2,2-diphenylacetoxy)piperidine-1-carboxylate Dihydrochloride **23**

The title compound was synthesized according to General Chemistry Procedure **3** from **23i** to obtain the title compound **23** (quantitative) as a white solid. ^1^H NMR (400 MHz, D_6_-DMSO) δ 8.76 (1H, dd, *J* = 8.7, 2.7 Hz), 8.17 (3H, s), 7.80 (3H, s), 7.43–7.22 (10H, m), 6.81–6.68 (1H, m), 6.63 (1H, s), 5.10–5.05 (1H, m), 4.21 (1H, 2d, *J* = 8.6 Hz), 3.95–3.90 (1H, m), 3.35–3.25 (4H, m), 2.76–2.74 (2H, m), 1.75–1.72 (4H, m), 1.65–1.31 (4H, m), 1.43 (3H, 2d, *J* = 8.6 Hz), 1.40–1.35 (2H, m), 0.93 (9H, 2s).^13^C (101 MHz, CDCl_3_) δ 172.76, 172.53, 169.61, 158.63, 143.81, 128.29, 127.95, 127.57, 90.59, 81.18, 70.54, 52.28, 38.97, 38.77, 34.60, 34.42, 31.32, 26.95, 26.81, 26.75, 21.45, 19.96; *m*/*z* (ESI; 99%) calcd for C_34_H_48_N_4_O_8_ = 640.78 found 641.2 [M + H]^+^.

##### (10*S*,13*S*)-10-((*tert*-Butoxycarbonyl)amino)-13-((*S*)-*sec*-butyl)-2,2-dimethyl-4,11,14-trioxo-3,15-dioxa-5,12-diazaheptadecan-16-yl-4-(2-hydroxy-2,2-diphenylacetoxy)piperidine-1-carboxylate **24i**

The title compound was synthesized according to General Chemistry Procedure **2** from **16** and *N*,*N*-bis(*tert*-butoxycarbonyl)-l-lysine and was purified using flash column chromatography using a gradient of 20–80% EtOAc:(40–60) PET to obtain the title compound **24i** as a mixture of diastereoisomers (94%) as a colorless oil. ^1^H (400 MHz; CDCl_3_) δ 7.47–7.30 (10H, m), 6.75 (1H, q, *J* = 5 Hz), 5.32 (4H, m), 5.20–5.10 (2H, m), 4.70 (1H, bs), 4.60–4.50 (1H, m), 4.83–4.64 (1H, m), 4.06 (1H, bs), 3.50–3.05 (6H, m), 1.95–1.75 (2H, m), 1.74–1.58 (3H, m), 1.56–1.52 (2H, m), 1.51–1.47 (3H, m), 1.46 (18H, s), 1.43–1.34 (2H, m), 0.95–0.84 (9H, m); 13C (101 MHz, CDCl_3_) δ 173.85, 171.20, 169.91, 156.19, 152.58, 141.88, 128.16, 127.37, 127.12, 90.48, 81.05, 79.14, 71.70, 56.31, 51.58, 41.35, 37.69, 29.69, 28.30, 27.67, 23.87, 22.62, 21.05, 19.72, 14.20, 11.58; *m*/*z* (ESI; 99%) calcd for C_44_H_64_N_4_O_12_ = 841.01 found 842.5 [M + H]^+^.

##### 1-((l-Lysyl-l-alloisoleucyl)oxy)ethyl-4-(2-hydroxy-2,2-diphenylacetoxy)piperidine-1-carboxylate Dihydrochloride **24**

The title compound was synthesized according to General Chemistry Procedure **3** from **24i** to obtain the title compound **24** (quantitative) as a white solid. ^1^H (400 MHz; D_6_-DMSO) δ 8.93 (1H, d, *J* = 6.9 Hz), 8.35 (3H, bs), 8.07 (3H, bs), 7.47–7.30 (10H, m), 6.68 (1H, q, *J* = 5 Hz), 5.09–5.02 (1H, m), 4.32–4.21 (1H, m), 3.76–3.67 (1H, m), 3.45–3.11 (4H, m), 2.76–2.70 (2H, m), 1.86–1.69 (4H, m), 1.64–1.46 (4H, m), 1.46–1.36 (7H, m), 1.30–1.18 (3H, m), 0.92–0.76 (6H, m); ^13^C (101 MHz, D_6_-DMSO) δ 172.74, 169.58, 169.53, 158.98, 143.76, 132.19, 132.05, 129.12, 128.24, 127.93, 127.51, 90.39, 81.17, 67.87, 57.25, 51.92, 38.71, 38.67, 36.60, 30.85, 30.26, 26.69, 25.13, 25.05, 19.96, 15.57, 11.68; *m*/*z* (ESI; 99%) calcd for C_34_H_48_N_4_O_8_ = 640.78 found 641.5 [M + H]^+^.

##### (10*S*,13*S*)-10-((*tert*-Butoxycarbonyl)amino)-13-isopropyl-2,2-dimethyl-4,11,14-trioxo-3,15-dioxa-5,12-diazaheptadecan-16-yl-4-(2-hydroxy-2,2-diphenylacetoxy)piperidine-1-carboxylate **25i**

The title compound was synthesized according to General Chemistry Procedure **2** from **14** and *N*,*N*-bis(*tert*-butoxycarbonyl)-l-lysine and was purified using flash column chromatography using a gradient of 20–80% EtOAc:(40–60) to obtain the title compound **25i** as a mixture of diastereoisomers (95%) as a colorless oil. ^1^H (400 MHz; CDCl_3_) δ 7.45–7.30 (10H, m), 6.76 (1H, q, *J* = 5 Hz), 5.32 (4H, m), 5.27 (1H, bs), 4.86–4.74 (1H, bs), 4.53–4.37 (1H, m), 3.57–3.00 (6H, m), 2.19–2.08 (1H, m), 1.84–1.72 (3H, m), 1.67–1.55 (3H, m), 1.53–1.48 (2H, m), 1.47–1.43 (3H, d, *J* = 5 Hz), 1.41 (18H, s), 1.37–1.30 (2H, m), 0.93–0.84 (6H, m); ^13^C (101 MHz; CDCl_3_) δ 173.74, 172.23, 171.15, 156.18, 152.55, 141.91, 128.08, 127.34, 90.51, 81.05, 79.94, 71.54, 60.36, 56.83, 41.31, 40.94, 40.42, 33.81, 31.55, 31.09, 29.58, 28.27, 27.62, 23.90, 18.86, 18.68; *m*/*z* (ESI; 97%) calcd for C_43_H_62_N_4_O_12_ = 826.99 found 827.5 [M + H]^+^.

##### 1-((l-Lysyl-l-valyl)oxy)ethyl-4-(2-hydroxy-2,2-diphenylacetoxy)piperidine-1-carboxylate Dihydrochloride **25**

The title compound was synthesized according to General Chemistry Procedure **3** from **25i** to obtain the title compound **25** (quantitative) as a white solid. ^1^H (400 MHz; D_6_-DMSO) δ 8.83 (1H, d, J= 7.1 Hz), 8.35 (3H, bs), 8.06 (3H, bs), 7.45–7.30 (10H, m), 6.68 (1H, q, *J* = 5 Hz), 5.08–5.02 (1H, m), 4.48–4.10 (2H, m), 3.39–3.17 (3H, m), 2.80–2.70 (2H, m), 2.13–2.03 (1H, m), 1.84–1.72 (2H, m), 1.80–1.68 (2H, m), 1.67–1.55 (2H, m), 1.65–1.54 (2H, m), 1.53–1.46 (2H, m), 1.45–1.34 (4H, m), 0.96–0.85 (6H, m); ^13^C (101 MHz; D_6_-DMSO) δ 172.75, 171.34, 169.76, 158.59, 152.54, 143.76, 128.26, 127.94, 127.51, 117.77, 114.87, 90.33, 81.17, 70.39, 51.93, 38.71, 30.84, 30.03, 26.73, 26.69, 21.39, 19.99, 19.19; *m*/*z* (ESI; 97%) calcd for C_33_H_46_N_4_O_8_ = 626.75 found 627.4 [M + H]^+^.

##### (10*S*,13*R*)-10-((*tert*-Butoxycarbonyl)amino)-13-isopropyl-2,2-dimethyl-4,11,14-trioxo-3,15-dioxa-5,12-diazaheptadecan-16-yl-4-(2-hydroxy-2,2-diphenylacetoxy)piperidine-1-carboxylate **26i**

The title compound was synthesized according to General Chemistry Procedure **2** from **19** and *N*,*N*-bis(*tert*-butoxycarbonyl)-l-lysine and was purified using flash column chromatography using a gradient of 20–80% EtOAc:(40–60) PET to obtain the title compound **26i** as a mixture of diastereoisomers (92%) as a colorless oil. ^1^H (400 MHz; CDCl_3_) δ 7.30–7.45 (10H, m), 6.76 (1H, q, *J* = 5 Hz), 5.32 (4H, m), 5.27 (1H, bs), 4.74–4.86 (1H, bs), 4.37–4.53 (1H, m), 3.00–3.57 (6H, m), 2.08–2.19 (1H, m), 1.72–1.84 (3H, m), 1.55–1.67 (3H, m), 1.48–1.53 (2H, m), 1.43–1.47 (3H, d, *J* = 5 Hz), 1.41 (18H, s), 1.30–1.37 (2H, m), 0.84–0.93 (6H, m); ^13^C (101 MHz, CDCl_3_) δ 173.74, 172.23, 171.15, 156.18, 152.55, 141.91, 128.08, 127.34, 90.51, 81.05, 79.94, 71.54, 60.36, 56.83, 41.31, 40.94, 40.42, 33.81, 31.55, 31.09, 29.58, 28.27, 27.62, 23.90, 18.86, 18.68; *m*/*z* (ESI; 99%) calcd for C_43_H_62_N_4_O_12_ = 826.99 found 827.5 [M + H]^+^.

##### 1-((l-Lysyl-d-valyl)oxy)ethyl-4-(2-hydroxy-2,2-diphenylacetoxy)piperidine-1-carboxylate Dihydrochloride **26**

The title compound was synthesized according to General Chemistry Procedure **3** from **26i** to obtain the title compound **26** (quantitative) as a white solid. ^1^H (400 MHz; D_6_-DMSO) δ 8.9 (1H, dd, *J* = 8.2, 2.1 Hz), 8.4 (3H, s), 8.1 (3H, s), 7.4–7.2 (10H, m), 6.7 (1H, q, *J* = 5.9, 5.4 Hz), 4.2 (1H, ddd, *J* = 8.3, 5.8, 2.9 Hz), 3.9 (1H, d, *J* = 10.8 Hz), 3.4–3.1 (4H, m), 2.7 (2H, *J* = 6.3 Hz), 2.1 (1H, td, *J* = 13.8, 7.2 Hz), 1.8 (4H, ddt, *J* = 13.8, 9.3, 4.0 Hz), 1.7–1.5 (4H, m), 1.4 (3H, d, *J* = 5.4 Hz), 1.3–1.2 (2H, m), 0.9 (6H, tq, *J* = 6.0, 3.9, 3.2 Hz).^13^C (101 MHz, D_6_-DMSO) δ 172.73, 169.94, 169.63, 152.55, 143.77, 132.07, 129.13, 128.26, 127.94, 127.51, 90.54, 81.17, 67.88, 57.83, 52.16, 31.09, 30.43, 30.26, 28.83, 26.63, 23.72, 22.86, 21.54, 20.00, 19.96; *m*/*z* (ESI; 97%) calcd for C_33_H_46_N_4_O_8_ = 626.75 found 627.4 [M + H]^+^.

##### (6*S*,9*S*)-9-(4-((*tert*-Butoxycarbonyl)amino)butyl)-6-((*S*)-*sec*-butyl)-2,2-dimethyl-4,7,10-trioxo-3,11-dioxa-5,8-diazatridecan-12-yl-4-(2-hydroxy-2,2-diphenylacetoxy)piperidine-1-carboxylate **27i**

The title compound was synthesized according to General Chemistry Procedure **2** from 1-((*N*_6_-(*tert*-butoxycarbonyl)-l-lysyl)oxy)ethyl 4-(2-hydroxy-2,2-diphenylacetoxy)piperidine-1-carboxylate, and (*tert*-butoxycarbonyl)-l-isoleucine and was purified using flash column chromatography using a gradient of 20–80% EtOAc:(40–60) PET to obtain the title compound **27i** as a mixture of diastereoisomers (49%) as a colorless oil. ^1^H (400 MHz; CDCl_3_) δ 7.48–7.30 (10H, m), 6.69 (1H, q, *J* = 5 Hz), 5.21–5.10 (2H, m), 5.62–5.11 (1H, m), 4.38–4.34 (1H, m), 3.94 (1H, bs), 3.48–2.97 (6H, m), 1.90–1.78 (3H, m), 1.73–1.62 (3H, m), 1.57–1.51 (2H, m), 1.51–1.46 (3H, d, *J* = 5 Hz), 1.44 (9H, s), 1.40–1.30 (2H, m), 0.96–0.87 (8H, m); ^13^C (101 MHz; CDCl_3_) δ 173.81, 171.68, 170.29, 156.08, 152.65, 141.84, 128.14, 127.35, 90.75, 82.71, 81.05, 71.64, 59.23, 51.84, 40.91, 40.46, 36.99, 36.96, 31.57, 29.79, 29.21, 28.46, 28.31, 24.76, 23.90, 19.73, 17.30, 14.66; *m*/*z* (ESI; 99%) calcd for C_44_H_64_N_4_O_12_ = 841.01 found 841.5 [M + H]^+^.

##### 1-((l-Isoleucyl-l-lysyl)oxy)ethyl-4-(2-hydroxy-2,2-diphenylacetoxy)piperidine-1-carboxylate Dihydrochloride **27**

The title compound was synthesized according to General Chemistry Procedure **3** from **27i** to obtain the title compound **27** (quantitative) as a white solid. ^1^H (400 MHz; D_6_-DMSO) δ 8.84 (1H, t, *J* = 6.6 Hz), 8.26 (3H, bs), 7.95 (3H, bs), 7.48–7.30 (10H, m), 6.63 (1H, q, *J* = 5 Hz), 5.08–5.03 (1H, m), 4.27–4.18 (1H, m), 3.48–3.11 (4H, m), 2.69–2.80 (2H, m), 1.90–1.78 (4H, m), 1.87–1.62 (3H, m), 1.57–1.51 (2H, m), 1.60–1.47 (5H, m), 1.46–1.36 (4H, m), 0.96–0.80 (6H, m); ^13^C (101 MHz; D_6_-DMSO) δ 174.59 172.75, 168.51, 161.04, 143.77, 128.27, 127.95, 127.51, 81.18, 65.39, 56.64, 52.37, 40.68, 40.47, 38.73, 36.68, 30.18, 26.79, 19.96, 15.64, 11.64; *m*/*z* (ESI; 97%) calcd for C_34_H_48_N_4_O_8_ = 640.78 found 641.8 [M + H]^+^.

##### (10*S*)-10-(4-((*tert*-Butoxycarbonyl)amino)butyl)-2,2-dimethyl-4,8,11-trioxo-3,12-dioxa-5,9-diazatetradecan-13-yl-4-(2-hydroxy-2,2-diphenylacetoxy)piperidine-1-carboxylate **28i**

The title compound was synthesized according to General Chemistry Procedure **2** from 1-((*N*_6_-(*tert*-butoxycarbonyl)-l-lysyl)oxy)ethyl 4-(2-hydroxy-2,2-diphenylacetoxy)piperidine-1-carboxylate and 3-((*tert*-butoxycarbonyl)amino)propanoic acid and was purified using flash column chromatography using a gradient of 20–80% EtOAc:(40–60) PET to obtain the title compound **28i** as a mixture of diastereoisomers (34%) as a colorless oil. ^1^H (400 MHz; CDCl_3_) δ 7.47–7.30 (10H, m), 6.73 (1H, q, *J* = 5 Hz), 5.30–5.09 (2H, m), 4.01 (1H, bs), 3.47–3.05 (6H, m), 2.48–2.33 (2H, m), 1.85–1.73 (3H, m), 1.71–1.58 (3H, m), 1.57–1.50 (2H, m), 1.50–1.44 (3H, d, *J* = 5 Hz), 1.41 (18H, s), 1.37–1.31 (2H, m); ^13^C (101 MHz; CDCl_3_) δ 173.73, 171.73, 170.68, 156.49, 152.73, 141.89, 134.40, 133.40, 129.64, 129.61, 129.46, 129.44, 129.34, 129.31, 128.12, 127.35, 126.88, 126.86, 90.92, 81.07, 79.28, 71.55, 63.83, 63.76, 52.01, 40.58, 38.60, 36.65, 35.98, 31.21, 29.75, 28.39, 23.93, 19.63; *m*/*z* (ESI; 99%) calcd for C_41_H_58_N4_3_O_12_ = 798.93 found 800.0 [M + H]^+^.

##### 1-(((3-Aminopropanoyl)-l-lysyl)oxy)ethyl-4-(2-hydroxy-2,2-diphenylacetoxy)piperidine-1-carboxylate Dihydrochloride **28**

The title compound was synthesized according to General Chemistry Procedure **3** from **28i** to obtain the title compound **28** (quantitative) as a white solid. ^1^H (400 MHz; D_6_-DMSO) δ 8.61 (1H, t, *J* = 7.1 Hz), 7.90 (6H, bs), 7.47–7.30 (10H, m), 6.62 (1H, q, *J* = 5 Hz), 6.60 (1H, bs), 5.08–5.03 (1H, m), 4.14–4.13 (1H, m), 3.41–3.15 (4H, m), 3.01–2.91 (2H, m), 2.76–2.66 (2H, m), 2.59–2.54 (1H, m), 1.83–1.70 (2H, m), 1.68–1.46 (6H, m), 1.45–1.31(6H, m); ^13^C (101 MHz, D_6_-DMSO) δ 172.75, 170.23, 152.64, 143.77, 128.28, 127.96, 127.51, 90.55, 81.18, 75.02, 61.63, 52.29, 38.82, 35.58, 32.20, 30.31, 26.90, 22.49, 20.00; *m*/*z* (ESI; 99%) calcd for C_31_H_42_N_4_O_8_ = 598.70 found 599.8 [M + H]^+^.

### DMPK: General Methods

All animal studies were ethically reviewed and carried out in accordance with Animals (Scientific Procedures) Act 1986 and the GSK Policy on the Care, Welfare, and Treatment of Animals.

#### Lung Homogenate Assay

Whole lung from Wistar Han rat, obtained from Charles River Laboratories, was taken after euthanasia, weighed (approximately 1.5 g), and placed into a 15 mL Precellys 24 Dual Evolution homogenizing vessel containing ceramic beads. The lung was prehomogenized at 2 °C for 5 cycles of 20 s at 7400 rpm with 30 s intervals. The homogenate was then diluted 4 times by mass with HPLC grade water (approximately 6 mL) and then homogenized once more under the same conditions.

Binding assays were determined in RED plates purchased from Thermofisher. Rat lung homogenate was either created on the day or stored at at least −20 °C for a maximum of one freeze–thaw cycle. Whole rat blood was taken in-house and used on the day of assay, storing at 4 °C if necessary. RED plates were prepared by placing the spiked matrix (100 μL, 1000 ng/mL) [rat lung homogenate] prepared in a t-vial into the first six RED ring chambers. The unspiked matrix (100 μL) was added to the final two RED chambers. Dialysis buffer (pH 6.5 phosphate-buffered saline, 300 μL) was added to the top six buffer chambers, and the bottom two buffer chambers remained empty to calculate recovery. The RED plate was sealed with a sealant tape and masking tape and incubated at 37 °C for 4 h on an orbital shaker. Once the RED plate was prepared, the spiked matrix from the original t-vial (20 μL, 1000 ng/mL) was added to a labeled micronic immediately followed by control dialysis buffer (20 μL) and internal standard (300 μL, 6.25 ng/mL labetalol in MeCN and 17.5 ng/mL reserpine in MeCN), creating the time 0 sample. After 4 h, the RED plate was removed from the incubator, unsealed, and the spiked matrix from the original t-vial (20 μL, 1000 ng/mL) was added to a labeled micronic immediately followed by a control dialysis buffer (20 μL) and internal standard (300 μL, 6.25 ng/mL labetalol in MeCN and 17.5 ng/mL reserpine in MeCN), creating the time 240 sample. The RED plate was then sampled by removing 20 μL from each well into a labeled micronic tube. The incubated control matrix or incubated control PBS (20 μL) was added to the matrix match as follows: the incubated control PBS sample (20 μL) was added to the sample from the red ring (20 μL) in a labeled micronic tube in a 96-well plate or the incubated control matrix (20 μL) was added to the buffer sample from buffer wells (20 μL) in a labeled micronic tube in a 96-well plate. All samples consist of matrix:PBS 1:1. To each micronic tube was added internal standard (300 μL, 6.25 ng/mL labetalol in MeCN and 17.5 ng/mL reserpine in MeCN), and the plate was shaken for 10 min and centrifuged for a further 20 min. The plate was then submitted for mass-spec analysis, quantifying the relative prodrug/drug mass ion peak against that of the internal standard.

#### Prodrug Stability Assays

In triplicate, to a 37 °C, prewarmed aliquot of prodrug in D_6_-DMSO (5 μL, 200 μg/mL) in a plastic micronic tube in a 96-well plate was added the prewarmed stability assay matrix (995 μL) (either phosphate buffer (pH 6.5 or 7.4), rat lung homogenate or rat blood). The resulting solution (1 mL, 1000 ng/mL) was maintained at 37 °C and shaken continuously throughout the assay. At predetermined intervals, samples of the reaction mixture (20 μL) were diluted in the internal standard (300 μL, 6.25 ng/mL labetalol in MeCN and 17.5 ng/mL reserpine in MeCN), shaken for 10 min, and centrifuged for a further 20 min. The sample was then submitted for mass-spec analysis, quantifying the relative prodrug/drug mass ion peak against that of the internal standard.

#### Intratracheal Pharmacokinetic Studies

The in-life phase was performed at Saretius Ltd., under a Home Office license P513DA7FD. Compound **23** was formulated in solution (5% ethanol in pH 6.5 PBS) and dosed at 0.2 mg/kg with a dosing volume of 1 mL/kg to male rats (*n* = 3, Sprague Dawley supplied from Charles River Laboratories) via intratracheal administration. Serial blood samples were taken at 0.25, 0.5, 1, 2, 3, 5, 8, and 24 h. An anticoagulant was added (22 μL EDTA (93 mg/mL) per 1 mL of blood), and blood samples were held on ice before centrifugation for plasma (10,000 rpm × 3 min). At 24 h, lungs were harvested from the euthanized rats (CO_2_). The lungs were rinsed in saline, blot-dried, weighed, and snap-frozen in liquid N_2_. All samples were stored at −20 °C prior to mass-spec quantification, which was performed at Sygnature Discovery. Plasma samples were prepared by protein precipitation with methanol/acetonitrile containing the internal standard (Imipramine) under standard protocols. Lungs were homogenized, and the protein was precipitated with methanol/acetonitrile containing the internal standard (Imipramine). The LC–MS/MS method for sample quantification was a Thermo TSQ Quantivia with a Thermo Vanquish UPLC system, a Phenomenex Luna Omega 1.6 μm, C18 100 Å, 50 × 2.1 mm column. Solvent A Milli-Q water + 0.1% formic acid; solvent B methanol + 1% formic acid, flow rate 0.8 mL/min using a gradient of 0–99.9% B over 1.15 min., column temperature 65 °C.

## Supporting Information Available

Molecular formula strings (CSV)
Structural characterization of compounds **23** and **26**; ^1^H NMR studies on compounds **17** and **23** in D_6_-DMSO + pH 6.5 PBS (PDF)

## Abbreviations Used

°C: degrees Celsius
BOC: boc, *tert*-butoxycarbonyl
cLogP: calculated logP
cmpd: compound
DCM: dichloromethane
DMF: dimethylformamide
DMPK: drug metabolism and pharmacokinetics
ESI: electrospray ionization
EtOAc: ethyl acetate
HCl: hydrochloric acid
HPLC: high-performance liquid chromatography; high-pressure liquid chromatography
ITPK: intratracheal pharmacokinetics
LC–MS: liquid chromatography–mass spectrometry
MeCN: acetonitrile
MeOH: methanol
MS: mass spectrometry
NMR: nuclear magnetic resonance
PBS: phosphate-buffered saline
