# Supplementary material for: Design, Synthesis, and Evaluation of Lung-Retentive Prodrugs for Extending the Lung Tissue Retention of Inhaled Drugs
Source: J Med Chem. 2022 Jul 7;65(14):9802–18. doi: 10.1021/acs.jmedchem.2c00416 (PMC9340777; doi:10.1021/acs.jmedchem.2c00416)
Supplement: Supplementary file 2 — jm2c00416_si_002.pdf [file jm2c00416_si_002.pdf]

S1: Supporting Information

Design, synthesis and evaluation of lung retentive prodrugs for extending the lung tissue retention of inhaled drugs.

*Jack Ayre[1], Joanna M. Redmond[2], Giovanni Vitulli[2], Laura Tomlinson[2], Richard Weaver[3], Eleonora Comeo[1], Cynthia Bosquillon[4], Michael J. Stocks[1]**

[1] School of Pharmacy, Biodiscovery Institute, University Park Nottingham, Nottingham, NG7 2RD, UK.

[2] GSK Medicines Research Centre, Gunnels Wood Road, Stevenage, SG1 2NY, UK

[3] XenoGesis Ltd, Discovery Building, BioCity, Pennyfoot Street, Nottingham, NG1 1GR, UK

[4] School of Pharmacy, Boots Science Building, University Park Nottingham, Nottingham, NG7 2RD, UK.

*Corresponding Author: michael.stocks@nottingham.ac.uk

S1 Structural characterization of compound **23**

LCMS purity trace

^{1}H NMR in d6-DMSO

S2 ^{1}H NMR/LCMS studies on compound **23** in d6-DMSO + pH 6.5 PBS

S3 ^{1}H NMR studies on compound **17** in d6-DMSO + pH 6.5 PBS

S4 ^{1}H NMR of compound **26** in d6-DMSO

S1 Structural characterization of compound **23**

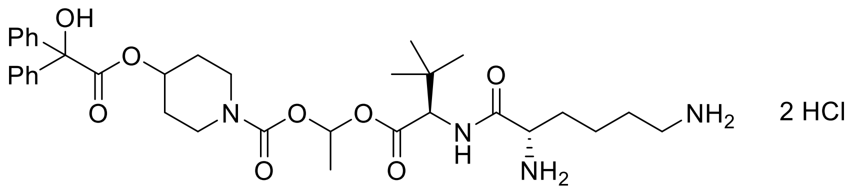

LCMS purity and molecular weight trace:

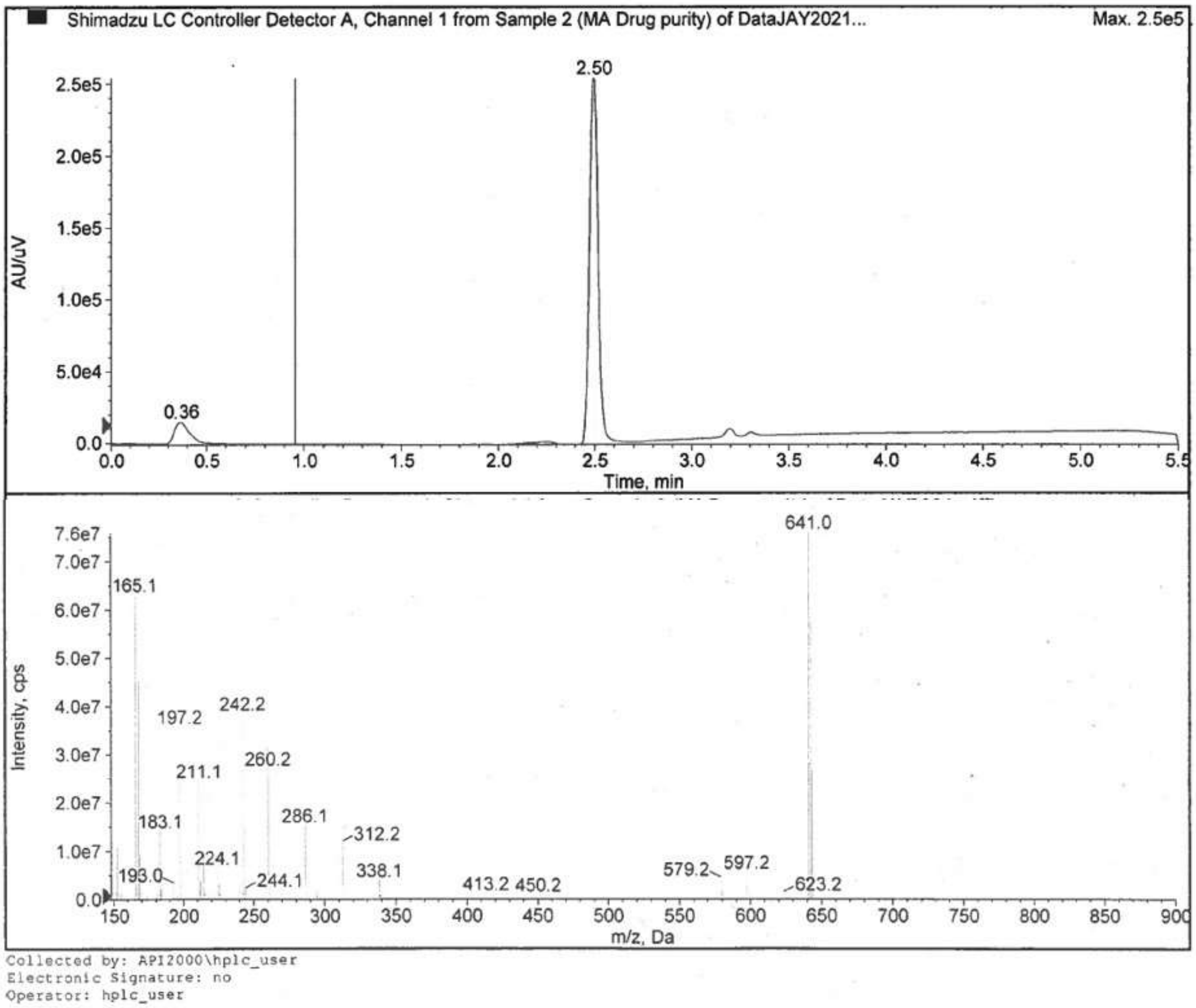

^{1}H NMR characterization of compound **23**

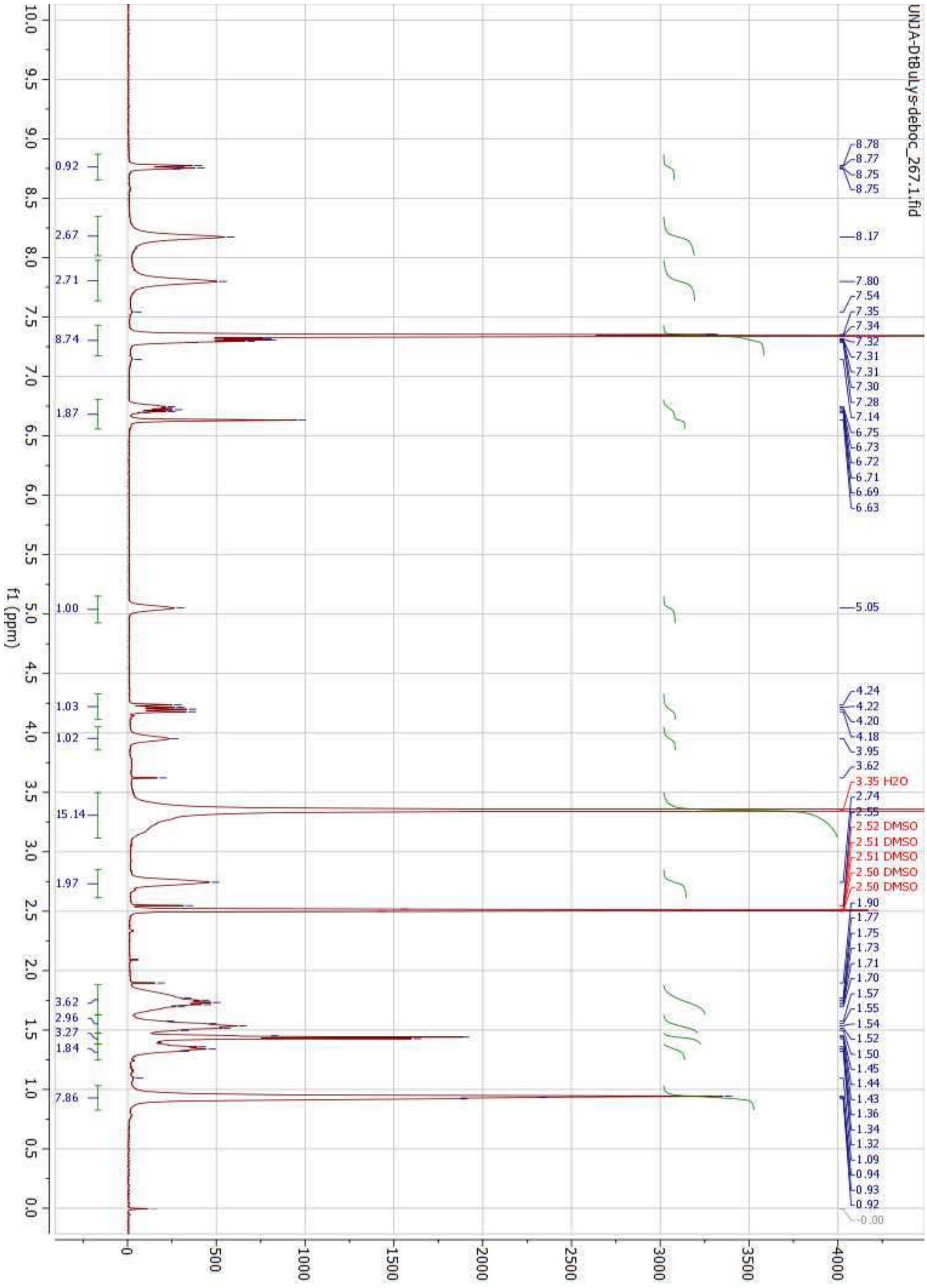

^{1}H NMR (400 MHz, D6-DMSO) δ 8.76 (1H, dd, J = 8.7, 2.7 Hz), 8.17 (3H, s), 7.80 (3H, s), 7.43 – 7.22 (10H, m), 6.81 – 6.68 (1H, m), 6.63 (1H, s), 5.10-5.05 (1H, m), 4.21 (1H, 2d, J = 8.6 Hz), 3.95-3.90 (1H, m), 3.35-3.25 (4H, m), 2.76-2.74 (2H, m), 1.75-1.72 (4H, m), 1.65 – 1.31 (4H, m), 1.43 (3H, 2d, J = 8.6 Hz), 1.40-1.35 (2H, m), 0.93 (9H, 2s) ppm.

S2 ^{1}H NMR studies on compound **23** in d6-DMSO + pH 6.5 deuterated PBS

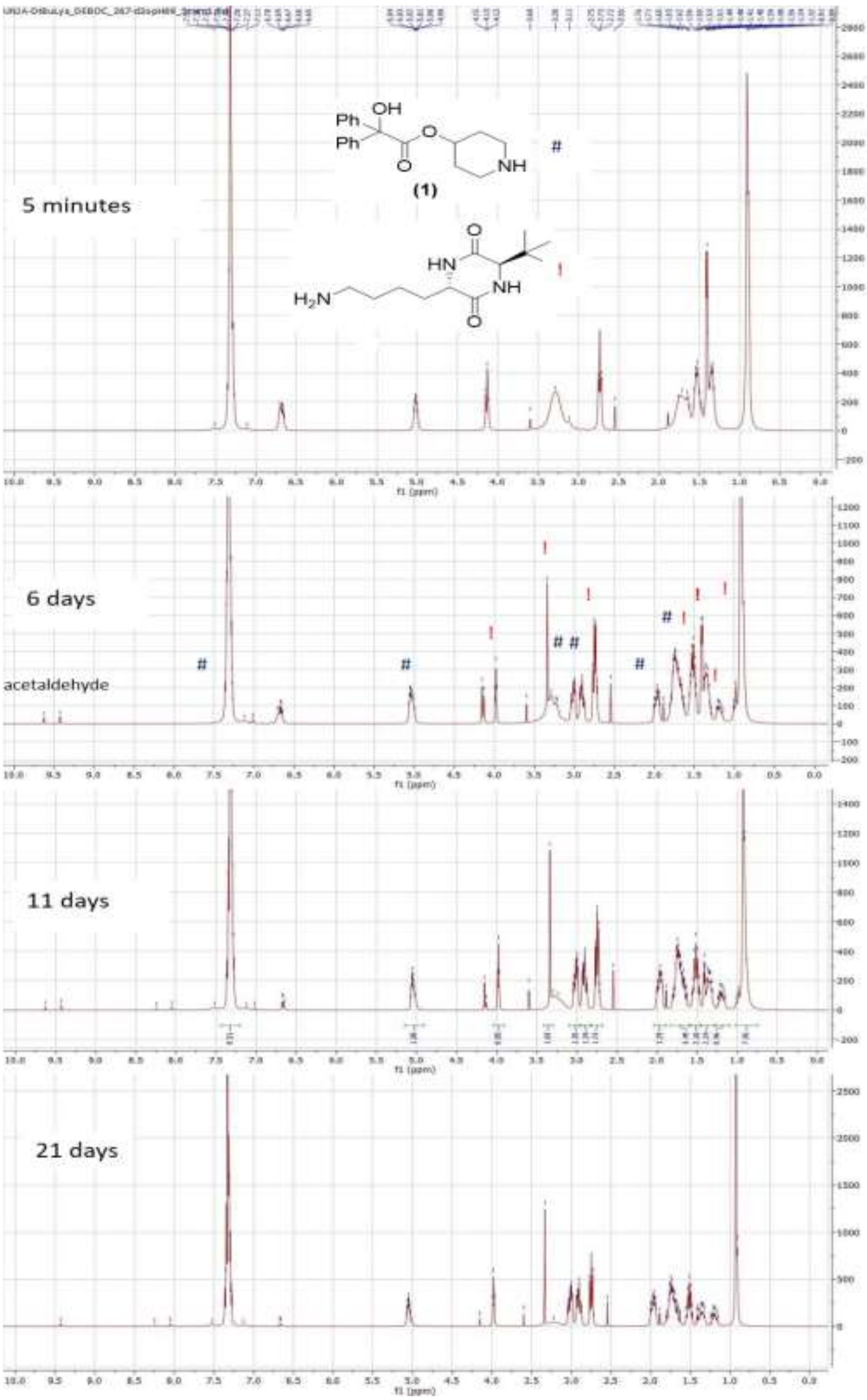

Figure S2 shows compound **23** at T=0 hours, T = 6, 11 and 21 days with the addition of pH6.5 deuterated PBS. (D_2O and DMSO signals suppressed)

LCMS analysis of compound **23** in d6-DMSO + pH 6.5 deuterated PBS

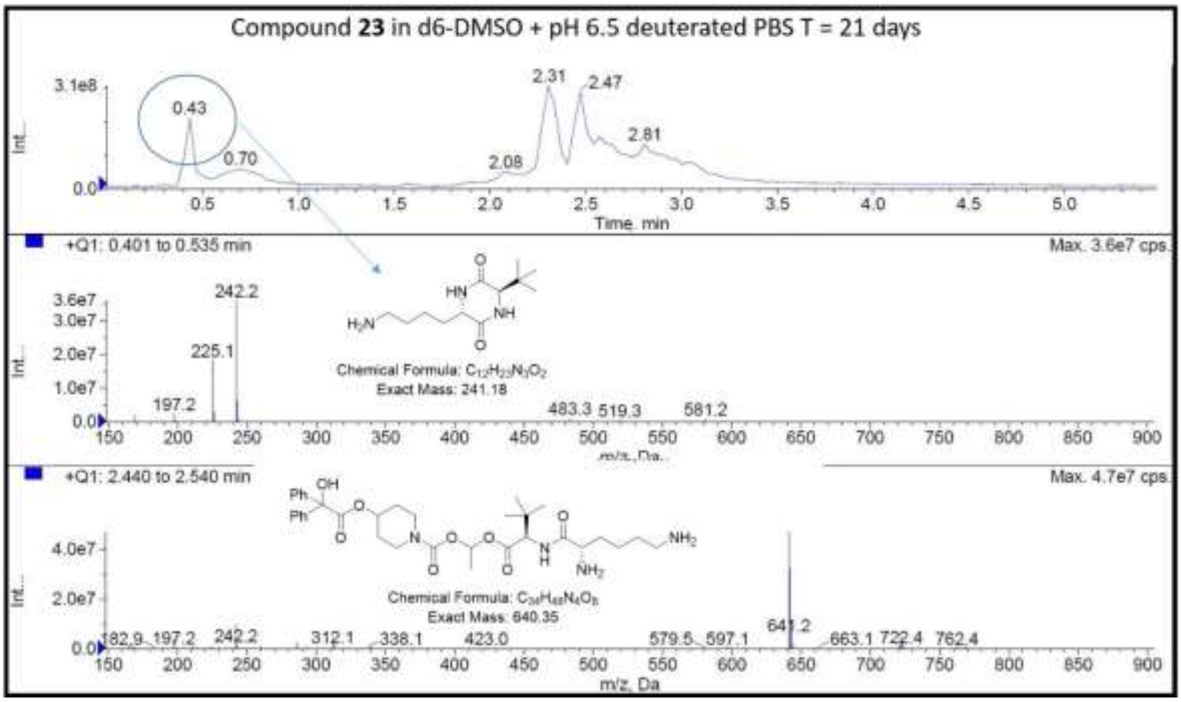

Compound **23** at T= 21 days with the addition of pH6.5 deuterated PBS. Diketopiperazine observed by LCMS at T=0.43 mins. Residual prodrug 23 observed at T=2.47 mins.

S3 ^{1}H NMR studies on compound **17** in d6-DMSO + pH 6.5 deuterated PBS

A time course ^{1}H NMR experiment performed to show the breakdown of compound **17**, using deuterated pH 6.5 PBS. Figure S3 shows compound **17** at T=0 hours (D) and the same sample at 48 hours (C). Clean conversion to breakdown products are clearly shown as the active drug (**1**, A)), lysine (B), acetaldehyde and ethanol.

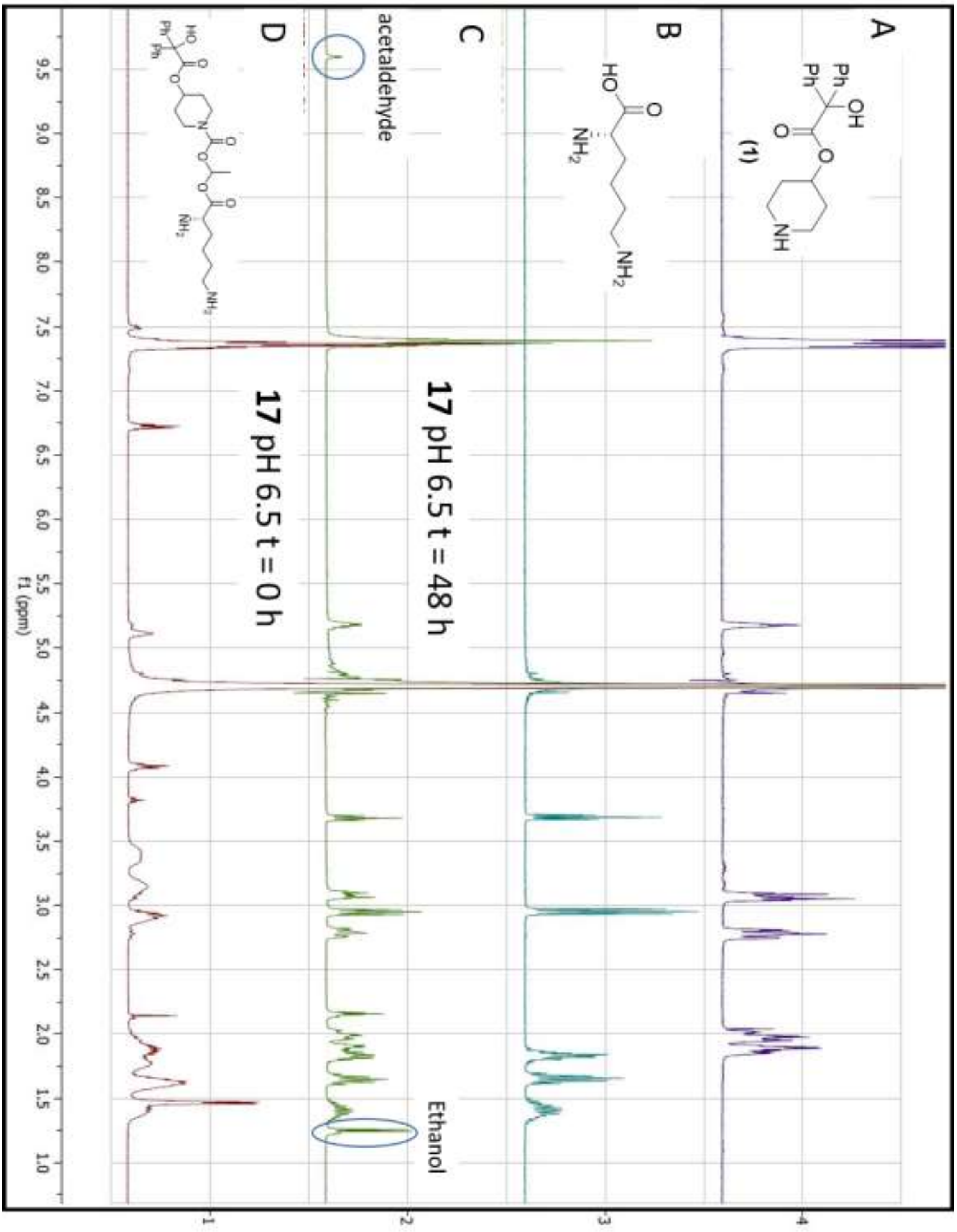

S4 ^{1}H NMR of compound **26** in d6-DMSO (DMSO solvent peaks suppressed). The tertiary alcohol was not visible in this spectrum.

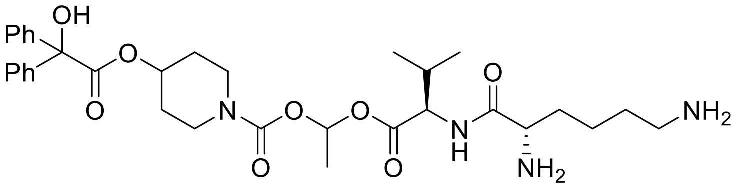

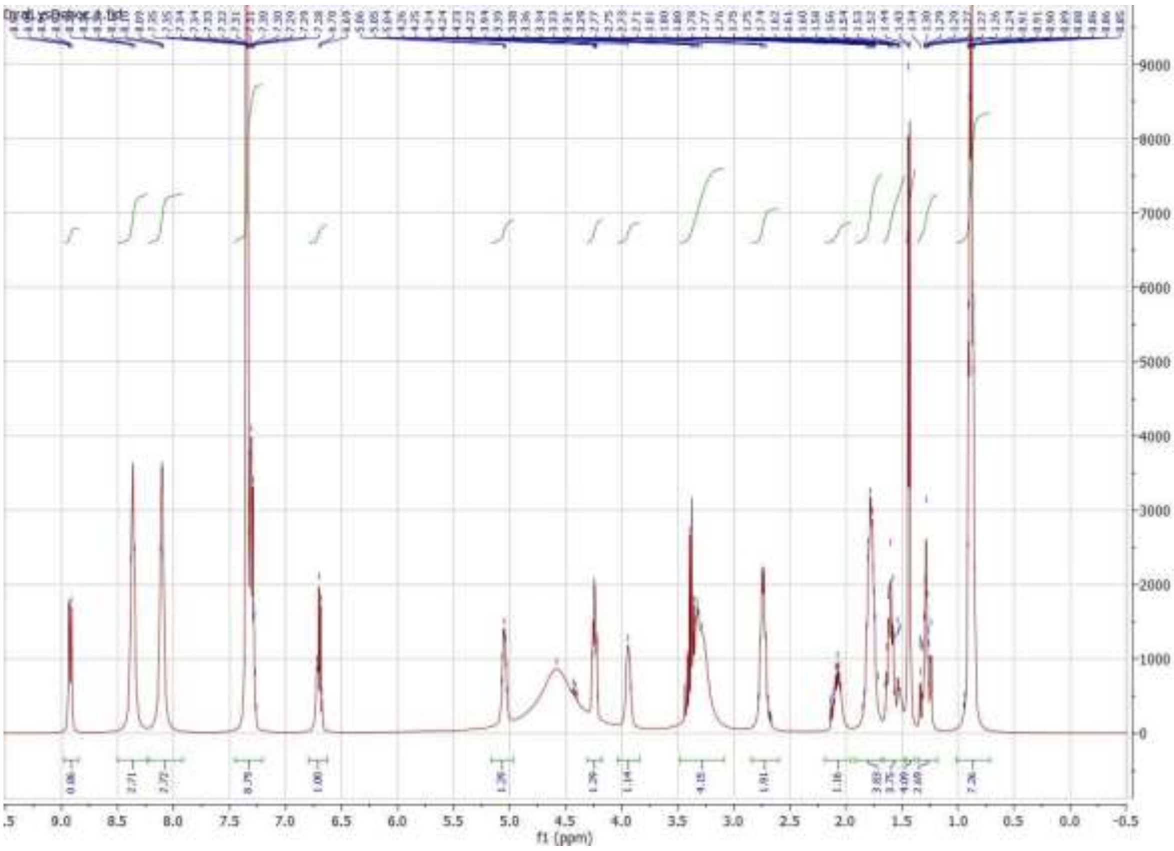

^{1}H NMR (400 MHz, DMSO-d6) δ 8.9 (1H, dd, J = 8.2, 2.1 Hz), 8.4 (3H, s), 8.1 (3H, s), 7.4 – 7.2 (10H, m), 6.7 (1H, q, J = 5.9, 5.4 Hz), 4.2 (1H, ddd, J = 8.3, 5.8, 2.9 Hz), 3.9 (1H, d, J = 10.8 Hz), 3.4 – 3.1 (4H, m), 2.7 (2H, J = 6.3 Hz), 2.1 (1H, td, J = 13.8, 7.2 Hz), 1.8 (4H, ddt, J = 13.8, 9.3, 4.0 Hz), 1.7 – 1.5 (4H, m), 1.4 (3H, d, J = 5.4 Hz), 1.3 – 1.2 (2H, m), 0.9 (6H, tq, J = 6.0, 3.9, 3.2 Hz) ppm.
